# Distributional impact of the Malawian Essential Health Package

**DOI:** 10.1093/heapol/czaa015

**Published:** 2020-05-03

**Authors:** Matthias Arnold, Dominic Nkhoma, Susan Griffin

**Affiliations:** c1 Inav, Berlin, Germany; c2 Health Economics and Policy Unit, College of Medicine, University of Malawi, Lilongwe, Malawi; c3 Centre for Health Economics, University of York, York, UK

**Keywords:** Health benefits package, distributional cost-effectiveness analysis, low- and middle-income country, Malawi, priority setting, health equity

## Abstract

In low- and middle-income countries (LMICs), making the best use of scarce resources is essential to achieving universal health coverage. The design of health benefits packages creates the opportunity to select interventions on the basis of explicit objectives. Distributional cost-effectiveness analysis (DCEA) provides a framework to evaluate interventions based on two objectives: increasing population health and reducing health inequality. We conduct aggregate DCEA of potential health benefits package interventions to demonstrate the feasibility of this approach in LMICs, using the case of the Malawian health benefits package. We use publicly available survey and census data common to LMICs and describe what challenges we encountered and how we addressed them. We estimate that diseases targeted by the health benefits package are most prevalent in the poorest population quintile and least prevalent in the richest quintile. The survey data we use indicate socioeconomic patterns in intervention uptake that diminish the population health gain and inequality reduction from the package. We find that a similar set of interventions would be prioritized when impact on health inequality is incorporated alongside impact on overall population health. However, conclusions about the impact of individual interventions on health inequalities are sensitive to assumptions regarding the health opportunity cost, the utilization of interventions, the distribution of diseases across population groups and the level of aversion to inequality. Our results suggest that efforts to improve access to the Essential Health Package could be targeted to specific interventions to improve the health of the poorest fastest but that identifying these interventions is uncertain. This exploratory work has shown the potential for applying the DCEA framework to inform health benefits package design within the LMIC setting and to provide insight into the equity impact of a health benefits package.

**Key Messages**

- Health benefits packages that move countries towards universal health coverage can be designed to improve population health and equity.
- The application of formal quantitative methods to examine value for money and distributional impacts can be challenging in settings with limited and fragmented health data.
- Using the case study of the Malawian Essential Health Package, we demonstrate the application of an aggregate distributional cost-effectiveness framework to support the prioritization of health interventions into a health benefits package.

## Introduction

Recent examples from low and middle-income countries (LMICs) in Africa (Todd *et al*., 2016) and Latin America (Giedion *et al.*, 2014) demonstrate the use of health benefits packages as a means of focusing scarce resources on interventions that provide the best value for money (Glassman *et al.*, 2016). The National Health Policy in Malawi (2017–22) documents the Malawi Government’s aim to move towards universal health coverage of its Essential Health Package (EHP). The redesign of the EHP in 2017 was informed using cost-effectiveness analyses (Ochalek *et al.*, 2018) that provide information on the cost per disability adjusted life year (DALY) averted of potential interventions. When combined with an estimate of the DALYs that could be averted with alternative uses of health sector funds, this allows interventions to be ranked based on the net DALYs averted. This is useful if the objective is to maximize population health benefits, i.e. DALYs averted, from the available budget.

In Malawi, as in many LMICs, the intention is that the health benefits packages address another key consideration, namely equity in health, healthcare access and use. The burden of ill health is greatest in the poorest, as indicated in Malawi by the higher rate of infant mortality, stunting, underweight, diarrhoea and respiratory disease in poorer groups compared to richer (Zere *et al.*, 2007). This inequality is exacerbated by disproportionately higher utilization of healthcare among the rich (Zere *et al.*, 2007). Both the Malawi National Policy and Health Sector Strategic Plan identify the reduction of health inequalities as a goal (Zere *et al.*, 2007; Umuhoza and Ataguba, 2018). However, equity was considered informally in the process of designing the EHP, due to the lack of evidence on the health inequality impacts of interventions.

Using the Malawian EHP as an example, this article explores the potential for providing information on the health inequality impacts of interventions, in settings characterized by incomplete or fragmented national data systems. Such information would allow for the selection of interventions into a health benefits package based on their ability to increase population health and to reduce health inequality. It could also be used to show the overall impact of the health benefits package on health inequality and the potential value of eliminating inequality in access, and of achieving full coverage.

We apply aggregate-level distributional cost-effectiveness analysis (DCEA) (Love-Koh *et al*., 2019), using publicly available data sources, to potential EHP interventions. In essence, we aim to determine how much of the total population net health benefit from each intervention would fall to different equity relevant population groups, defined by area of residence and wealth. This would allow an assessment of how interventions change the distribution of health across those groups (Asaria *et al.*, 2015; Dawkins *et al.*, 2018). We show how an explicit value judgement, about how much society values increase in population health compared to reduction in health inequality, can be used to rank interventions where there are trade-offs. We highlight the challenges and assumptions required to perform this analysis for Malawi and suggest methods to overcome them.

## Materials and methods

### Data sources

We use a database of cost-effectiveness evidence that was established to inform the design of the Malawian EHP (Ochalek *et al.*, 2018). The database included intervention cost and health effects from the Tufts Global Health Cost-Effectiveness Registry, WHO Choice and systematic reviews (Ochalek *et al.*, 2018) and local epidemiological estimates of the size of the eligible population for each intervention (Ochalek *et al.*, 2016). Complete information was available for 73 interventions (Ochalek *et al.*, 2016), of which 51 are included in the current EHP.

For national estimates of total population size, age and gender distribution, as well as the proportions living in urban and rural areas, we use the preliminary results of the 2018 Malawi Population and Housing Census (National Statistical Office, 2018). We use estimates of the marginal productivity of the health service in Malawi of one DALY averted per $61 USD (Ochalek *et al.*, 2016) additional expenditure.

To estimate socioeconomic distributions of mortality rates, disease and healthcare utilization, we use information from the Demographic Household Survey (DHS) 2015–16, the Integrated Household Survey (IHS) 2016–17 and the Multiple Indicator Cluster Survey (MICS) 2013–14. These surveys are typical of the evidence available in settings such as Malawi and record socioeconomic information alongside disease burden and care seeking behaviour.

The IHS captures self-reported disease occurrence by asking an open question ‘During the last 2 weeks, did you suffer from an illness or injury?’ followed by the specification ‘What was the illness or injury?’. These questions are accompanied by questions about care seeking: ‘Who diagnosed the disease?’ and ‘What actions did you take to find relief for your illness?’. The answers have categories such as ‘I sought treatment at gov. health facility, church-based facility, village health clinic/health surveillance assistant’. This allows us to identify service utilization with the providers of the EHP. For example, in Malawi, the anticipated EHP providers are governmental and church-based care facilities. The DHS and MICS have a narrower focus than the IHS but add information on aspects of preventive care, nutrition and sexual health not captured in the IHS. The DHS includes questions about the age at death of respondent’s offspring and siblings. All three provide information on respondent’s age, gender, household composition, education status, housing and asset ownership.

The Global Burden of Disease Study (GBD) informs the overall level of health in terms of life expectancy and disease burden. For Malawi, GBD mortality estimates are based on several waves of the DHS, the Malawi Diffusion and Ideational Change Project, the Malawi Malaria Indicator Survey, the MICS, the Malawi Population and Housing Census, the Malawi Population Change Survey and the Malawi Family Formation Survey 1984. GBD uses 86 data sources for cause of death estimates and 155 data sources of non-fatal health outcomes [Institute of Health Metrics (IHME), 2018]. For each condition, GBD provides years of life lost to disability for the whole population, or in rates per 100 000. The GBD does not provide information on the differences between socioeconomic groups.

### Methods

We focus on the reduction in inequality in health-adjusted life expectancy (HALE) and the inequality associated with two socioeconomic characteristics: household wealth and urban vs rural residence. We therefore estimate distributions of health by stratifying the population into two subgroups based on residence and five subgroups based on a wealth asset index. We use the International Wealth Index to provide a common asset index across the surveys (Smits and Steendijk, 2015). The International Wealth Index assigns a score to each survey respondent that ranges from 0 (possesses none of the assets) to 100 (possesses all of the assets). By dividing the population into quintiles based on this index, we thus describe five subgroups of the population ranked by their asset ownership. We label these subgroups ‘poorest’, ‘poorer’, ‘middle’, ‘richer’ and ‘richest’, describing their relative wealth position.

We determine how interventions alter the health of different groups in the population. Cost-effectiveness studies analyse how introducing a given intervention would alter healthcare costs and population health outcomes, compared to the status quo. For example, the introduction of Rotavirus vaccination would cost an additional $0.69 and avert an additional 0.14 DALYs per child under one, compared to providing no vaccination. Interventions influence health directly, by offering health benefits to recipients (e.g. 0.14 DALYs). They also influence health indirectly, through the opportunity cost of being unable to use the resources each commands (e.g. $0.69) for other purposes. The incremental cost that each intervention imposes on the health system displaces alternative investments, leading to opportunity costs in the form of foregone health benefits. The marginal productivity of health service expenditure describes the rate at which costs impose foregone health benefits (e.g. $0.69 divide $61 = 0.01 DALYs). These forgone benefits are accounted for when interventions are evaluated using net health benefit, which describes the incremental direct health benefit minus the health opportunity cost. The net health benefit of Rotavirus vaccination is 0.14–0.69/61 = 0.13 DALYs.

net health benefit=incremental health benefit-health opportunity cost.

An aggregate DCEA takes the average incremental costs and health benefits reported from existing cost-effectiveness studies and combines these with socioeconomic distributions of utilization and opportunity cost to estimate how the net health benefit from an intervention is divided among equity relevant subgroups within the population.

The socioeconomic distribution of incremental net health benefit that we estimate for each intervention is added to a baseline distribution of HALE. The level of inequality in the distribution of HALE with each intervention is compared to the level of inequality in the baseline distribution, to estimate the potential change in inequality from each intervention.

We use the estimated impacts of the 51 EHP interventions in our sample to represent the socioeconomic impacts that might be obtained with 1 year of implementation of the full EHP and to estimate the potential value of increasing uptake from current levels to the full eligible population.

In the following four sections, we describe in more detail the methods applied, the data requirements, which data sources are utilized in our example, and our assumptions:

1. the estimation of the distributional impact of interventions,
2. the estimation of the baseline health distribution,
3. measurement of inequality and priority setting and
4. sensitivity analyses.

#### Method Stage 1: net distributional impact of health interventions

To calculate the distribution of the direct health benefits, i.e. the direct changes in health experienced by the recipients of any intervention, we need to know the amount of health generated per use, and how many individuals in each group receive the intervention.

We use the reported incremental health benefit in the database of cost-effectiveness evidence to represent the direct benefit per use (i.e. the number of DALYs averted). We assume this is the same for each person receiving the intervention, regardless of whether the person is in a rural or urban residence, or whether they are asset rich or asset poor.

We use the IHS as the main source of information for the socioeconomic distributions of disease and healthcare utilization and add information from DHS and MICS for interventions that were not included in the IHS. For example, we use the DHS to identify the distribution of insecticide-treated bed-nets. A full description of how we match the survey questions to diseases and interventions is in the Supplementary Appendix Table S1.

The total size of the eligible population over a period of 1 year from the database of cost-effectiveness evidence is divided into socioeconomic groups, according to the distribution of prevalence we observe in the survey data. The overall number of eligible individuals is then multiplied by the proportion of the total survey reported disease accounted for by each socioeconomic group, to give the number of individuals who would benefit from the intervention in each socioeconomic group per year.

We estimate the uptake of each intervention in each socioeconomic group as the ratio of patients who report utilizing healthcare for the condition, over the total number of patients who report experiencing the disease. We assume that the socioeconomic pattern in self-reported care seeking reflects the socioeconomic pattern of EHP utilization. The eligible population in each socioeconomic group multiplied by the uptake in that group determines the numbers of individuals treated at current utilization patterns.

For missing information on the proportion of individuals in each socioeconomic group who are eligible for an intervention, we use the mean distribution of prevalence. For missing information on the proportion of individuals who use interventions for which they are eligible (missing utilization) in each socioeconomic group, we use the mean uptake in each socioeconomic group multiplied by prevalence to get utilization.

In the absence of information about the degree to which changes in healthcare expenditure would affect the health in each socioeconomic group, we base our estimate of the distribution of health opportunity cost on the crude socioeconomic distribution of healthcare utilization. We estimate the proportion of each socioeconomic subgroup who sought care at a governmental or church-based health provider (i.e. the facilities providing public health care) across all disease reported in the IHS. We assume that this distribution of average utilization is a reasonable proxy for the marginal distribution. That is, when interventions are introduced or displaced, the socioeconomic characteristics of the affected individuals match those observed in average utilization. In essence, this assumes that the socioeconomic distribution of health benefits, from increasing or reducing healthcare budgets, is in line with the distribution of healthcare utilization.

Box 1 illustrates each step of the calculations with a worked example. This shows how we convert the incremental costs (B) and incremental health benefit (C) reported for Rotavirus vaccination into a distribution of net health benefit, using the size of the eligible population (A), survey information (D, E, G) and estimating health opportunity cost at a rate of one DALY per $61.

#### Method Stage 2: baseline distribution of health

The baseline distribution of health describes how mortality and morbidity differ between socioeconomic groups without any of the EHP interventions. We calculate lifetables for each socioeconomic group by adjusting the age-specific mortality rates from GBD, according to the socioeconomic distribution observed in mortality rates among respondent’s siblings and children reported in the DHS (see Supplementary Table S2). The deaths occurred prior to 2015, and hence prior to the imposition of the revised EHP in 2017. We use respondent’s socioeconomic status as a proxy for that of their children and siblings. In the base case analysis, we combine deaths reported for respondent’s sons and daughters (deaths reported for ages 0–35 years) with deaths reported for respondent’s siblings (deaths reported across all ages) and estimate mortality rates in 5 year bands up to age 55. We combine age 55–75 into one group and do not include deaths reported at older than age 75. The Sullivan method (Sullivan, 1971; Jagger *et al.*, 2006) is used to calculate life expectancy from the constructed life tables. Life expectancy in each socioeconomic group is then adjusted for years lost due to disability (YLD) to generate HALE. We use the YLD rates for each age group reported in the GBD. We match 19 diseases from GBD to one of 15 self-reported diseases across the DHS, IHS and MICS (Supplementary Table S3). The socioeconomic distribution in these 15 linked diseases is then used to proxy the distribution of the total YLD burden. Detailed descriptions of these calculations are found in the Supplementary Material.

#### Method Stage 3: measurement of inequality

We assess the impact of interventions on inequality in HALE based on the change in the Atkinson index. The Atkinson index measures relative inequality in a distribution, with weights for individuals determined by their level of health and the strength of aversion to health inequality. From the Atkinson index, we can determine the equally distributed equivalent (EDE) level of health, which is the amount of health that, if provided equally to everyone within a population, has the same worth as the current distribution (Asaria *et al.*, 2016). The EDE increases with the total amount of health and reduces with the degree of inequality in the distribution of health. The difference between the EDE level of health and the average population health can be interpreted as the cost of health inequality. The formula for EDE based on an Atkinson index is:

$$h_{\text{EDE}}={\left[\frac{1}{n}\overset{n}{\underset{i=1}{\sum }}h_{i}^{\left(1-\varepsilon \right)}\right]}^{\left(\frac{1}{1-\varepsilon }\right)},$$

where *ε* is the inequality aversion parameter, *h_i_* is the health of individual *i* and *n* is the total number of individuals in the population. In the absence of an estimate from Malawi, we use a starting value for the inequality aversion parameter of 10 (Robson *et al.*, 2017). Although the strength of aversion may differ depending on the nature of the inequality, to demonstrate our results in the base case, we apply this same value to represent aversion against inequality between wealth quintiles and between urban/rural residences.

We compare how introduction of an intervention would change the, EDE to how it would change overall population health. Interventions that increase population health and reduce health inequality will have an EDE impact greater than the net health impact, while interventions that increase health but increase health inequality will increase EDE by less than the amount they increase population health. We use this difference between EDE impact and net health benefit to describe the value of interventions’ impact on health inequality in terms of DALYs averted. We present our results in the health equity impact plane—a graph showing incremental population net health benefit on the *y* axis and inequality impact on the *x* axis (Cookson *et al.*, 2017).

#### Method Stage 4: sensitivity analyses

A summary of the key assumptions is given in Box 2. We apply extensive sensitivity analyses to test alternative assumptions for the distribution of the direct benefits of interventions, the health opportunity cost, the baseline distribution of health and the level of inequality aversion.

Sensitivity Analysis 1: two alternative estimates for the socioeconomic distribution of direct health benefits were defined. One scenario assumes equal prevalence across wealth quintiles. The second reflects a more unequal distribution by wealth quintile, where we increase prevalence for the poorest and poorer quintiles by 10% and decrease the prevalence for the richer and richest quintiles by 10%. In both scenarios, we retain the original uptake pattern.

Sensitivity Analysis 2: we explore the impact of different uptake patterns and retain the original prevalence distribution. One scenario assumes that all wealth quintiles have the population average level of uptake and a second scenario increased uptake in the poorest and poorer wealth quintiles by 10% and reduces uptake in the richer and richest quintiles by 10%.

Sensitivity Analysis 3: we vary the marginal productivity of the health service (i.e. the rate at which changes in expenditure generate health opportunity cost) between one DALY per additional $37 (higher opportunity cost) and one DALY per additional $116 (lower opportunity cost), on the basis of the range of empirical estimates for the region (Ochalek *et al.*, 2015; Woods *et al.*, 2016). In addition, we test two scenarios reflecting different distributions of opportunity across the wealth quintiles. We test a scenario where each wealth quintile bears an equal of 20% of the health opportunity cost and a more unequal scenario where we increase the proportion of the opportunity cost borne by the poorest and poorer quintiles by 10% and reduce the proportion borne by the richer and richest quintiles by 10%.

Sensitivity Analysis 4: for the baseline distribution of health, we test if the results change when the socioeconomic distribution of HALE is informed by the socioeconomic pattern of deaths that respondents report for their sons and daughters, up to age at the death of 20 years only.

Sensitivity Analysis 5: we vary the inequality aversion parameter to 2 (reflecting lower aversion to inequality) and 25 (reflecting very strong aversion to inequality).

## Results

### Socioeconomic profile of EHP-targeted diseases

**Figure 1:**
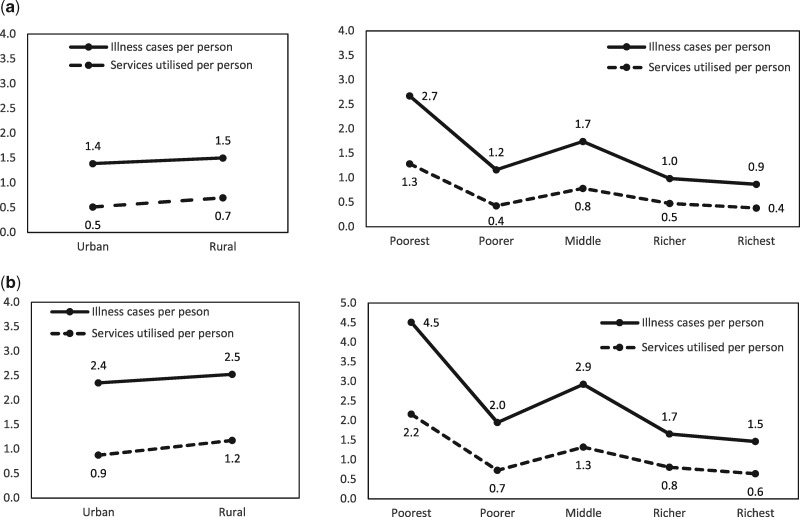
(a) Cases of illness and health services used per person (before imputation). (b) Cases of illness and health services used per person (after imputation).

Table 1 shows the population estimates, disease burden, service utilization and proportion of the health opportunity cost from changes in public healthcare expenditure for each socioeconomic group. Malawi has a population of ∼17 million, of whom 85% live in rural areas and 15% live in urban areas (National Statistical Office, 2018). There are 44 million cases of illness that qualify for treatment with one of the 51 EHP interventions reported in the health sector strategic plan (Government of the Republic of Malawi, 2017). Out of 44 million cases, we estimate that health services were utilized for 20 million, giving average uptake of health services of 45%. We estimate that, of the 20 million episodes of care with EHP interventions, 11% are to individuals from urban households and 89% to individuals from rural households. Alternatively, by wealth quintile, the utilization of EHP falls 38%, 13%, 23%, 14% and 11% as you move from the poorest to the richest households. Our base case estimate for the distribution of the health opportunity cost is that 17% of forgone health benefits are among individuals from urban households and 83% are for individuals from rural households. By wealth quintiles, the distribution is 23%, 22%, 22%, 20% and 19% from poorest to richest. In other words, for every one DALY that could be averted by expanding general health service expenditure, 0.83 DALYs would be averted among the rural population and 0.17 among the urban population. Figure 1 shows that, per person, disease burden and healthcare utilization broadly reduce by wealth quintile, and rates across rural and urban individuals are similar.

**Table 1:** Population, diseases and health services by socioeconomic group in millions

	Total population, *n*	Residence		Wealth quintiles				
		Rural, *n* (% of pop)	Urban, *n* (% of pop)	Poorest, *n* (% of pop)	Poorer, *n* (% of pop)	Middle, *n* (% of pop)	Richer, *n* (% of pop)	Richest, *n* (% of pop)
Population size	17.5	14.9 (85)	2.6 (15)	3.5 (20)	3.5 (20)	3.5 (20)	3.5 (20)	3.5 (20)
Disease cases (prevalence)	43.9	37.8 (86)	6.9 (14)	15.8 (36)	6.9 (16)	10.3 (23)	5.8 (13)	5.1 (12)
Health service utilized (utilization)	19.9	17.6 (88)	2.2 (11)	7.6 (38)	2.6 (13)	4.6 (23)	2.8 (14)	2.2 (11)
	Pop. average, %	% of disease cases	% of disease cases	% of disease cases	% of disease cases	% of disease cases	% of disease cases	% of disease cases
Uptake (services/ diseases)	45	47	37	48	37	45	49	44
	Total	% of total opp. cost	% of total opp. cost	% of total opp. cost	% of total opp. cost	% of total opp. cost	% of total opp. cost	% of total opp. cost
Opportunity cost	1 DALY per $61	83	17	23	22	20	19	16

### Intervention impact on net health and equity

**Figure 2:**
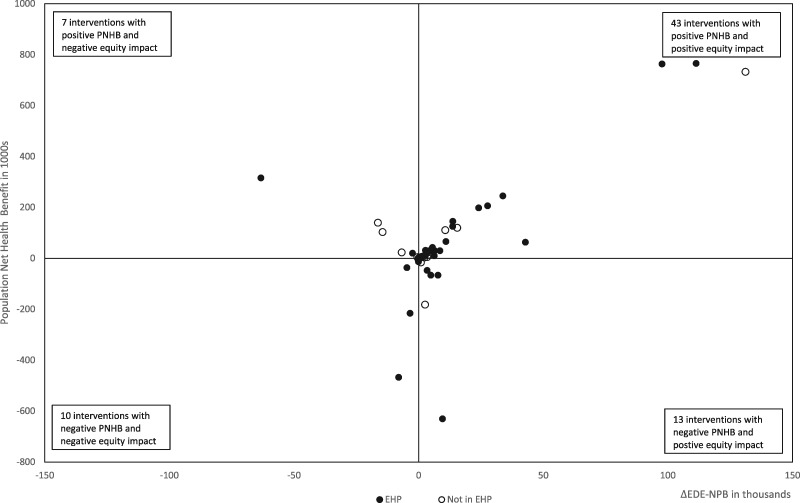
Equity plane, outliers ignored.

Figure 2 shows the 73 potential EHP interventions on the health equity impact plane, according to their impact on health inequality by wealth index. The underlying data for Figure 2 are provided in Table S5 in the Supplementary Appendix.

We find interventions in all four quadrants of the plane. Out of 43 interventions that increase population net health benefit and reduce health inequality, 36 (84%) are in the current EHP. Of 13 interventions for which we estimate negative population benefit but reductions in health inequality, 8 (62%) are included in the EHP. Of the seven interventions with positive population net health benefit and that increase health inequality, 3 (43%) are in the EHP. Out of 10 interventions with negative population net health benefit, and that increase health inequality, 4 (40%) are in the EHP.

### EHP impact across socioeconomic groups

**Figure 3:**
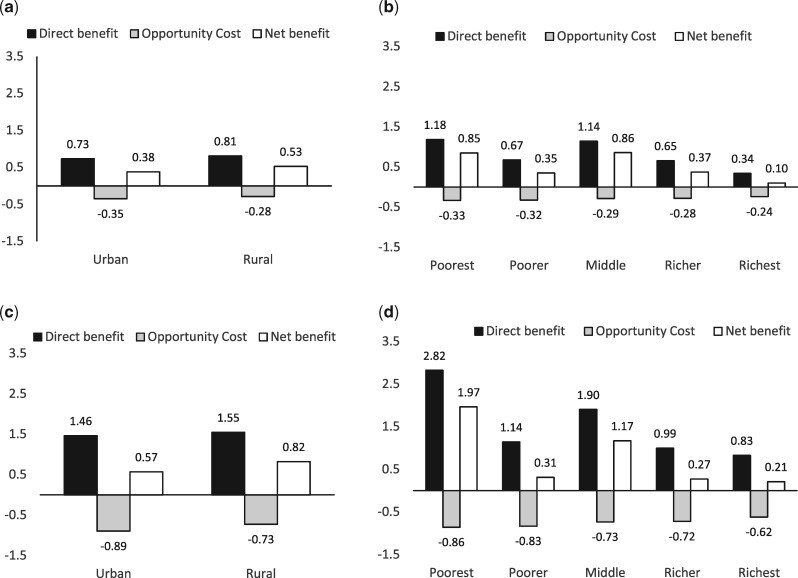
(a) Direct benefit, opportunity cost and net benefit* with expected service utilization by residence. (b) Direct benefit, opportunity cost and net benefit* with expected service utilization by wealth quintile. (c) Direct benefit, opportunity cost and net benefit* with full service utilization by residence. (d) Direct benefit, opportunity cost and net benefit* with full service utilization by wealth quintile. *Direct benefits, opportunity cost and net benefits are measured in averted DALY.

Figure 3 illustrates the socioeconomic distribution of impacts of the 51 interventions in the current EHP at current levels of health service utilization (Figure 3a and b), and if utilization were increased to 100% (Figure 3c and d). Of the 51 EHP interventions, 39 have positive incremental net health benefit (Supplementary Table S5).

Due to variation in the types of intervention used in each socioeconomic group, the direct health benefits from the EHP are very heterogeneous. Supplementary Table S4 shows the distribution of utilization across socioeconomic groups, and the incremental direct benefits for each intervention, allowing a comparison of the average direct health benefit across the interventions used in each socioeconomic group and the potential direct health benefit of services not used due to low uptake. The poorest households currently use EHP interventions that avert 1.18 DALYs but underutilize EHP interventions that could avert an additional 1.64 DALYs on average. Poorer households avert 0.67 DALYs with their current patterns of use and underutilize services that could avert on average 0.47 DALYs. The corresponding figures of DALYs averted with current patterns of use are 1.14 for middle wealth households, 0.65 for richer households and 0.34 for the richest households. The forgone DALYs averted due to underutilization are 0.76 for middle wealth households, 0.34 for richer households and 0.49 for the richest households.

We estimate that, on average, the EHP averts 0.51 DALYs per person in Malawi. This is composed from 0.53 DALYs averted per individual from rural households and 0.38 DALYs averted per individual from urban households. Alternatively, by wealth quintile, it is composed from 0.85 DALYs averted for each individual from the poorest wealth quintile, 0.35 DALYs averted per individual from the poorer quintile, 0.86 from the middle, 0.37 from the richer and 0.10 from the richest. Though the direct health benefits are highest for individuals in the poorest households, those individuals also carry the highest opportunity cost.

If utilization is increased to 100%, urban households would benefit from 0.57 DALYs averted in total, an increase of 50% compared to current utilization, and the benefit to rural households would increase to 0.82 DALYs (Figure 2c), an increase of 55%. The poorest quintile would benefit the most from increased uptake, leading to net benefit of a 1.97 averted DALY per person, a gain of 127%. Similarly, the DALYs averted in the middle wealth quintile households would increase to 1.17, an increase of 36%. However, the other wealth quintiles stand to lose from increased coverage of the EHP. The increased health opportunity costs in these groups outweigh the gains in direct health benefits.

### Impact on distribution of HALE

**Figure 4:**
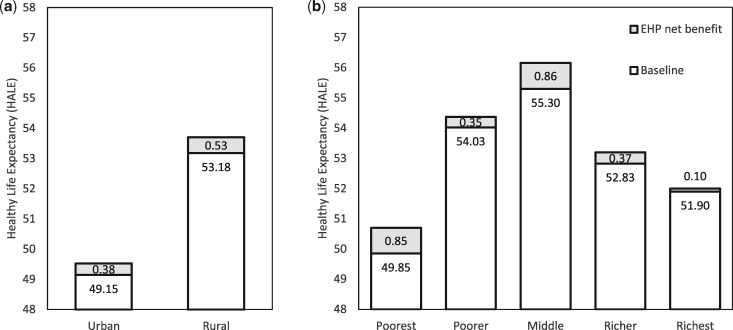
(a) Health-adjusted life expectancy by residence. (b) Health-adjusted life expectancy by wealth quintile.

Figure 4a and b illustrates how introduction of the EHP would change the distribution of HALE. Average baseline HALE is 49.15 years in urban residents and 53.18 years in rural households. Using the Atkinson index with an inequality aversion parameter of 10, this gives a baseline EDE HALE of 52.46 years compared to a baseline average HALE of 52.70, indicating a cost of inequality equal to 0.24 DALYs per person. The EHP increases HALE by 0.505 years on average, with 0.381 years for urban households and 0.526 healthy life years for rural households. The EHP improves the EDE HALE by 0.489 on average, leaving the cost of inequality in the post-EHP distribution equal to 0.26 DALYs per person.

By wealth quintile, baseline HALE varies between 49.85 (poorest) and 55.30 (middle).^1^ Given this baseline distribution, our use of an inequality aversion parameter of 10 results in an Atkinson index of 0.006. This implies a willingness to sacrifice 0.6% of total population health in Malawi, to eliminate inequality in HALE by wealth. The EHP increases HALE by 0.505 and EDE HALE by 0.516, indicating a reduction in inequality between wealth quintiles. At population level, the current 51 EHP interventions provide 8.87 million additional healthy life years, the EDE of which is 9.2 million healthy life years (given aversion to inequality in health by wealth quintile). The inequality impact is therefore equivalent in value to an additional 0.33 million DALYs averted (3.7% of the health benefits of the EHP).

### Priority ordering based on population net health benefit vs based on EDE

We compare prioritization of interventions by incremental net health benefit against ranking based on changes in EDE (Supplementary Table S5 in the Appendix). If all interventions with a positive incremental population net health benefit were selected, this would design a health benefits package of 50 interventions (out of 73 potential). If all interventions with positive change in EDE were selected, the health benefits package would include those same 50 interventions, and an additional 1 intervention with positive impact on EDE (high cholesterol treatment) but negative net health benefit.

### Sensitivity analysis

Table 2 provides an overview of the base case results and the sensitivity analyses considering health inequality by wealth quintile. Assuming equal distribution of disease reduces the estimated benefit from the 51 interventions in the current EHP. The estimated change in EDE health becomes lower than the change in population health benefit, suggesting that the EHP would increase the cost of inequality to 0.72 million DALYs. In contrast, assuming a more unequal disease burden doubles the estimated value of the EHP, in reducing inequality to 0.67 million DALYs averted compared to the base case (0.33 million DALYs averted).

**Table 2:** Sensitivity analyses

	Current 51 EHP interventions			Health equity plane position (NHB, EDE-NHB)				Out of 73 potential EHP interventions		
	ΔNHB	ΔEDE	DALY averted from inequality impact	Quadrant 1: ++	Quadrant 2: +−	Quadrant 3: −+	Quadrant 4: −−	NHB improving	EDE improving	Inequality (EDE-NHB) improving
Base case	8 872 286	9 204 107	331 821	43	7	13	10	50	51	56
Equal prevalence	8 681 387	7 958 206	−723 182	31	19	3	20	50	50	34
More unequal prevalence	8 887 783	9 561 023	673 240	43	7	13	10	50	51	56
Equal uptake	6 830 914	8 124 403	1 293 489	50	0	14	9	50	51	64
More unequal uptake	9 031 519	9 537 667	506 149	43	7	13	10	50	51	56
Opp. cost = $37/DALY	5 526 158	5 798 248	272 089	38	7	15	13	45	46	53
Opp. cost = $116/DALY	11 318 180	11 693 528	375 349	46	7	12	8	53	53	58
Equal opp. cost	8 872 286	9 262 551	390 265	43	7	15	8	50	51	58
More unequal opp. cost	8 872 286	9 018 293	146 008	43	7	6	17	50	51	49
Adjusting mortality only on basis of child mortality	8 872 286	9 604 957	732 672	48	2	6	17	50	51	54
Low inequality aversion (Atkinson *ε* = 2)	8 872 286	8 889 816	17 530	43	7	13	10	50	50	56
High inequality aversion (Atkinson *ε* = 25)	8 872 286	10 611 910	1 739 624	43	7	13	10	50	51	56

NHB: net health benefit; ΔNHB: change in net health benefit; ΔEDE: change in equally distributed equivalent.

**Table:** **Box 1**. Example calculations with Rotavirus vaccination

Rotavirus vaccination for children under 1						
Total population (A)	521 300					
Incremental health benefit (B)	0.14					
Incremental cost (C)	$0.69					
Total cost (A × C)	$809 318					
	Poorest	Poorer	Middle	Richer	Richest	Total
% survey reported cases of rotavirus (D)	36	16	23	13	12	100
DALYs averted if everyone vaccinated (A × B× D)	26 274	11 677	16 786	9488	8758	72 982
Uptake of vaccination (%) (E)	48	39	46	49	43	45
1. DALYs averted at current uptake (A × B × D × E)	12 611	4554	7721	4649	3766	33 302
Proportion of direct health benefit by subgroup	0.38	0.14	0.23	0.14	0.11	1
Cost by subgroup (A × C × E)	$172 655	$140 282	$165 461	$176 252	$154 670	$809 318
Proportion of opportunity cost by subgroup (F)	0.23	0.22	0.2	0.19	0.16	1
2. Health opportunity cost by subgroup [F × (A × C/61)]	3052	2919	2654	2521	2123	13 268
3. Net health benefit by subgroup (1–2)	9560	1635	5068	2128	1643	20 034
Proportion of net health benefit by subgroup	0.48	0.08	0.25	0.11	0.08	

Assuming equal uptake of interventions over all socioeconomic groups also reduces the estimated benefits of the EHP. Conversely, assuming a more unequal uptake, weighted in favour of the poorest and poorer, quintiles increases the amount by which the EHP is estimated to improve population health and EDE, increasing the estimated value of the reduction in health inequality with the EHP to 0.51 million DALYs averted.

Assuming equally distributed opportunity cost increases the amount by which the EHP is estimated to impact EDE health, while leaving the overall gain in population health unchanged. In contrast, assuming that health opportunity cost falls more heavily on the poorer and poorest quintiles reduces the amount by which the EHP is estimated to improve EDE health to 0.15 million DALYs averted.

Using only deaths for survey respondent’s offspring to inform the distribution of mortality provides an alternative distribution of baseline health by wealth quintiles. Healthy life expectancy is 50.32 years in poorest households, 51.82 in poorer households, 53.98 in middle households, 53.18 in richer households and 54.30 in richest households. This indicates a clearer association between wealth and HALE. Changing baseline health does not affect the estimated net population benefit from the EHP, but it does affect its impact on the EDE. Imposing the EHP on this alternative baseline distribution implies 0.73 million DALYs averted due to reduction in inequality.

For all scenarios, except those varying the level of the health opportunity cost and the level of health inequality aversion, the number of interventions estimated to reduce health inequality is sensitive to the alternative assumptions tested, but the number with overall positive impact on EDE is relatively insensitive. For these sensitivity analyses, the direction of the inequality impact and the magnitude of the gains from each intervention are affected, but not the conclusion about the set that should be prioritized for inclusion in a health benefits package when impacts on overall health and health inequality are combined.

Assuming health opportunity cost of one DALY averted per $37 reduces the estimated benefit of the EHP on both population health and health inequality. The value of the impact on inequality is reduced to 0.27 million DALYs averted. At this higher average health opportunity cost, only 45 interventions improve population health (averting 9.2 million DALYs) and 46 interventions improve EDE health. In contrast, assuming opportunity cost of one DALY averted per $116 increases the estimated impact of the EHP, leading to an additional gain of 0.38 million DALYs averted due to reduction in inequality. With lower health opportunity costs, 53 interventions would be estimated to improve population health (averting 13.5 million DALYs) and inequality. The set of interventions that would be prioritized differently, depending on the level of health opportunity cost, includes nine interventions (‘HIV interventions focused on female sex workers’, ‘HIV interventions focused on men who have sex with men’, ‘intermittent preventive therapy for pregnant women’, ‘high cholesterol treatment’, ‘management of moderate acute malnutrition in pregnant and lactating women’, ‘management of severe malnutrition in children’, ‘diarrhoea treatment with zinc’, ‘antibiotics for preterm premature rupture of membrane’ and ‘maternal sepsis case management’).

Assuming very low or very high aversion to inequality in health by wealth quintile leaves the amount by which the EHP is estimated to increase population health unaffected but alters its impact on the EDE health. However, the number of interventions estimated to increase EDE health is insensitive to both scenarios.

## Discussion

This is the first study to quantify the impact of a health benefits package on health inequality. Broadly, we found that there are data sources in LMICs that allow the estimation of socioeconomic distributions in disease prevalence, healthcare utilization and life expectancy. Using these, we could apply methods for aggregate DCEA. We found relatively little difference in overall net health benefits between urban and rural households, but that the EHP may be reducing differences in healthy life expectancy between wealth quintiles.

The current EHP was informed by cost-effectiveness analysis, but the selection of interventions was not based solely on whether they improve net population health. Our findings indicate that the probability of selection was higher for interventions that are health improving (78%—39 out of 50) and for those that we estimate to reduce inequality (79%—44 out of 56), compared to those that reduce population health (52%—12 out of 23) or that reduce inequality (41%—7 out of 17). Our study builds upon the earlier evaluation of interventions in Malawi by Ochalek *et al.* (2018) who focused on identifying best buys, and how health system constraints limit the implementation of the EHP. These health system constraints included factors on the demand side (such as lack of perceived benefits and difficulties in access) as well as factors on the supply side (such as lack of equipment or staff, supply chain bottlenecks, water and power shortages). Our analysis of survey information confirms that service use is suboptimal, with 45% uptake on average. Ochalek *et al.* find that the value of increasing coverage of the EHP outweighs the benefits from extending the package to include additional health promoting services. Our study adds to this by showing the potential reduction in health inequality, if uptake of health services is increased.

The results indicate how potential health package interventions differ in their impact on health inequality and population net health benefit. The survey data provide an indication that the diseases targeted by the Malawian EHP are most common in the poorest households. At the same time, we find that the number of EHP services utilized is highest in poorest households. However, they do not gain the most from current patterns of EHP access. We found that the selection of health interventions used by the poorest households averts fewer DALYs, on average, than the selection of interventions used by households in the middle wealth quintile. Interventions with high direct health benefits, such as active management of third stage of labour, first-line tuberculosis treatment and management of obstructed labour, remain underutilized by the poorest households. We did not explore the demand and supply side barriers that lead to this selection, but our results might indicate priority areas for future research to explain whether it is ameliorated by expanding access to the EHP. If full utilization of the EHP could be realized, poorest households would stand to gain the most.

Our results describe the distributional impact of the current EHP in the context of any current barriers to implementation and utilization. They therefore favour interventions that are more accessible to the poor, in addition to interventions for conditions that are more prevalent among the poor. Health service interventions that address demand and supply side barriers to the implementation of the EHP would influence the distributional impact of the EHP and the set of optimal interventions we identify. For example, introducing user fees for some services might reduce utilization disproportionately among the lower wealth quintiles and make those services less likely to reduce health inequality. However, the revenue generated by user fees may allow for the expansion of the EHP. The methods we propose here, and our results on current utilization, could assist in designing user fees to optimize their impact on health inequality.

Our study has limitations. We relied on survey information and a series of assumptions to estimate socioeconomic distributions of mortality, morbidity, direct benefits and foregone health.

We ranked households according to their relative position in the wealth distribution. Malawi is, however, one of the poorest countries in the world. The socioeconomic gradient between the poorest and richest households is comparatively slight. Even among the richest quintile, only 60% have access to electricity and 40% only have access to low quality water. Being rich in Malawi does not guarantee a high standard of living, and there may not be strong aversion to health inequality associated with wealth. Since the purpose of this study was to demonstrate the feasibility of DCEA, we did not yet consult policymakers in Malawi as to which socioeconomic characteristics best describe the nature of their health inequality concern. Future applications should be informed by the values of those bearing legitimate authority for determining healthcare resource allocation.

Self-reported disease might be systematically biased. This is a known problem in Malawi, especially in relation to rural communities and sensitive information (Baird and Ozler, 2012), diseases involving stigma (Bignami-Van Assche *et al.*, 2007) or interviewees answering according to the social desirability (Kelly *et al.*, 2013). The mortality rates reported in the DHS are for the respondent’s siblings and children. While socioeconomic characteristics do correlate within families and between siblings, the socioeconomic characteristics of the respondent are not necessarily the same as those of their siblings or children (Solon *et al.*, 1991; Heflin and Pattillo, 2006).

For future applications, cohort studies and initiatives such as the INDEPTH network might provide better information on socioeconomic differences in health (INDEPTH Resource & Training Centre, 2019). However, while changes in the baseline socioeconomic pattern of healthy life expectancy affected our conclusions about the overall equity impact of the EHP, the prioritization of different interventions for inclusion in a health benefits package was not affected.

We made a set of strong assumptions, to which we applied a series of sensitivity analyses to test how changes to distributional patterns in mortality, disease prevalence, health service uptake, opportunity cost and the overall level of opportunity cost and inequality aversion affected our conclusions. We found that changing the distributional patterns affects the magnitude and, in the case of the prevalence of disease, the direction by which the EHP is estimated to change health inequality. However, the selection of interventions to include in a health benefits package was relatively insensitive. We found that only the level of health opportunity cost has substantial impact on the potential size of the health benefits package. One assumption we did not address was that of equal direct health benefit from a given intervention, regardless of the individual’s socioeconomic characteristics. If wealthier individuals have greater capacity to benefit from interventions, we will have overestimated the direct health benefits to the poorest and potentially overestimated the reduction in health inequality from the EHP. However, given the mild wealth gradient in Malawi, it is unlikely that differential efficacy in wealth quintiles would affect our results.

Overall, we find that it is feasible to use aggregate DCEA to support the design of health benefits packages in two ways: first, by providing a structure for assessing the distributional impact of interventions and thus enabling formal deliberation on inequality impacts and, second, by providing a rational approach for evaluating any trade-off between impacts on health inequality and overall population health. The data used in this study are publicly available and thus this analysis could be replicated in other LMICs.

## Conclusion

Our analysis finds that the Malawian EHP includes interventions which target diseases that occur disproportionately among the poorest households. However, its impacts on improving healthy life expectancy and reducing health inequality are limited by socioeconomic patterns in underutilization. The DCEA framework allowed us to estimate the impact of each intervention on health inequality and to demonstrate that the set of health services used by the poorest households is comparatively less beneficial than those used by richer households. Despite facing several challenges when conducting DCEA in a data constrained setting such as Malawi, we found the DCEA framework to be a feasible and transparent method to explore the equity impact of health benefits packages in LMICs.

## Supplementary data

Supplementary data are available at *Health Policy and Planning* online.

**Box 2.** Summary of key assumptions

- The socioeconomic status of survey respondents is a suitable proxy for the socioeconomic status of their children and siblings (or for children only in Sensitivity Analysis 4).
- The socioeconomic distribution of self-reported disease in the last 2 weeks is a suitable proxy for the socioeconomic distribution of disease prevalence (varied in Sensitivity Analysis 1).
- The socioeconomic distribution of self-reported care seeking for each disease is a suitable proxy for the socioeconomic distribution of utilization of EHP interventions for that disease (varied in Sensitivity Analysis 2).
- The direct health benefit of each person receiving an intervention is the same regardless of socioeconomic status, i.e. we assume equal efficacy of interventions across socioeconomic groups.
- The socioeconomic pattern in care seeking across all diseases reported in the IHS describes the pattern of use of healthcare services in Malawi (varied in Sensitivity Analysis 3).
- The socioeconomic distribution in utilization of health services represents the socioeconomic distribution that would be observed for marginal changes in healthcare provision (Varied in Sensitivity Analysis 2).

## Supplementary Material

czaa015_Supplementary_Data

## Footnotes

1 As health-adjusted life expectancy is a non-linear weighted function of the age-specific rates, we get a slight discrepancy when disaggregating by wealth quintiles (52.78) vs residence (52.59).
