# Erratum: Distributional impact of the Malawian Essential Health Package

**DOI:** 10.1093/heapol/czaa059

**Published:** 2020-05-25

**Authors:** Matthias Arnold, Dominic Nkhoma, Susan Griffin

**Affiliations:** c1 Inav, Berlin, Germany; c2 Health Economics and Policy Unit, College of Medicine, University of Malawi, Lilongwe, Malawi; c3 Centre for Health Economics, University of York, York, UK

*Health Policy and Planning*, doi: 10.1093/heapol/czaa015

In the original article, there was an error in the published title. At typesetting the "i" in impact was accidently removed. The corrected version now displays the full word "impact" in the title. A typographical error was also removed on page 9. The Publisher apologizes for this error.

