# Supplementary material for: Distributional impact of the Malawian Essential Health Package
Source: Health Policy Plan. 2020 May 3;35(6):646–56. doi: 10.1093/heapol/czaa015 (PMC7294245; doi:10.1093/heapol/czaa015)
Supplement: czaa015_Supplementary_Data [file czaa015_supplementary_data.docx]

**Distributional impact of the Malawian EHP – Supplementary Appendix**

# Section 1: Calculating the distribution of baseline health

The baseline distribution of health gives the reference point for health improvements. The interventions in a health benefits package work in two dimensions, by extending length of life or by improving quality of life. One measure which includes these two aspects is the health-adjusted life-expectancy (HALE). We follow a WHO recommendation of using the Sullivan method (Jagger et al., 2006, Sullivan, 1971) by adjusting life expectancy from life table calculation by years lost due to disability (YLD) to estimate HALE (WHO, 2014). These life tables and YLD estimates need to be specific to each socio-economic group.

### Distribution of life expectancy

Life tables can be generated with a variety of methods depending on the available data. If available, life tables can be generated from a vital information registry tracking deaths in the population. The vital registry needs to track the socio-economic characteristics of interest to allow a socio-economic clustering of mortality. Since this requirement is very often not fulfilled or vital information are not available at all, alternative source of information are often utilised. Cohort studies or surveys may provide the required level of detail for a smaller proportion of the population.

Our approach to estimate life tables for socio-economic groups is to use the GBD to get age-specific mortality rates and adjust these mortality rates with the socio-economic distribution of self-reported mortality rates from the DHS. To estimate mortality rates by socioeconomic group we use respondents’ socioeconomic status as a proxy for that of their children and siblings (De Walque and Filmer, 2013). We combine observations and deaths from mortality in respondents’ children and siblings to create a combined dataset from which we estimate mortality rates. We apply the method for the calculation of adult mortality, which enables the estimation of mortality rates for 5-year age cohorts from the combined dataset of child and sibling mortality for all age cohorts (Croft et al., 2018). For age groups above 55 observations are sparse. We combine all age groups between age 55 and 75 into one, and do not estimate socio-economic differences in mortality above age 75. The number of deaths and total observations for both children and siblings are shown in the supplementary appendix Table S2.

By using the GBD mortality rates as a base and the DHS as socio-economic distributions, we get a baseline mortality profile that is derived from a multitude of surveys rather than only the latest DHS. For those years, where we can identify a socio-economic distribution, we use the DHS to adjust the GBD mortality rates using the following procedure: We adjust the age- -specific mortality rates in the GBD by the rate ratio of age-specific mortality rates between the socio-economic group and the general population from DHS. We thus use seven socio-economic sets (urban, rural, five wealth quintiles) of age-specific mortality ratios (from DHS) to calculate sets of mortality profiles, which can use then to calculate the life expectancy following the Sullivan method (Jagger et al., 2006, Sullivan, 1971). The adjustment can be described as this:

$${MR}_{SES}^{Age}={GBD\_MR}_{GeneralPopulation}^{Age}*\frac{{DHS_{MR}}_{SES}^{Age}}{{DHS_{MR}}_{GeneralPopulation}^{Age}}$$

The resulting mortality rates thus are specific for each age, gender and socio-economic group and can be used in addition with the population size in each group to calculate life expectancy.

### Distribution of YLD

To capture the loss in HALE due to morbidity, we use a similar approach of using age and gender-specific YLD rates (Jagger et al., 2006, Sullivan, 1971) from the GBD as a baseline and adjust them with socio-economic distributions from surveys. We use the following notation: We describe YLD population burden estimates with capitals indicating the subgroup selection in subscript, for example ${YLD}_{Urban}$ to describe the YLD burden for the urban population. We describe YLD rates (per 100.000 population) for specific diseases and subpopulations as ${YLDRate}_{subpopulation}^{Disease}$. Finally, we use small letters to describe the proportion of YLD in a specific subpopulation or disease, ${yld}_{SES}^{Disease}$. Our approach is to use disease-specific YLD rates for diseases from the GBD database, ${YLDrate}^{Disease i}$, and adjust these rates using distributions across age groups (from GBD), ${yld}_{Age}$, and socio-economic groups from the surveys, ${yld}_{SES}^{Disease i}$.

We use a method described by Dawkins et al. (2018) of using selected diseases accounting for the largest burden of disease. We identify 15 diseases representing over 50% of overall YLD, which link GBD diseases to survey responses in Integrated Household Survey (IHS), DHS and Multi-Indicator Cluster Survey (MICS). In one case, we applied the broader disease category of mental health to smaller diseases categories in the GBD (schizophrenia, bipolar disorder, anxiety, depression and other mental disorders) assuming that there socio-economic distribution is the same. The list of diseases and the mapping between GBD and surveys is shown in Table S2. From the surveys we get self-reported disease prevalence for each gender as a proportion of respondents who indicate they had the disease or received treatment for the disease. We calculate ratios of proportions between respondents in the socio-economic groups and the general population for each disease, ${yld}_{SES}^{Disease i}$. For example, the prevalence of Asthma in the general population is 1.47%, with 1.81% in urban and 1.39% in rural sides. We calculate the ratios of these proportions as ${yld}_{Urban}^{Asthma}=\frac{0.0181}{0.0147}=1.23$ and ${yld}_{Rural}^{Asthma}=\frac{0.0130}{0.0147}=0.95$.

1. ${yld}_{SES}^{Disease i}=\frac{{Proportion with disease}_{SES}^{Disease i}}{{Proportion with disease}_{General Population}^{Disease i}}$

We use these disease- and socio-economic-specific ratios, ${yld}_{SES}^{Disease i}$, to adjust the YLD rate of each disease in the socio-economic group, ${YLDrate}^{Disease i}$ and calculate the overall YLD burden with the respective population size in the socio-economic group, ${YLD}_{SES}$. For diseases that could not be linked, we applied a weighted average $\bar{yld}_{SES}^{All Diseases}$.

1. ${YLD}_{SES}={Population}_{SES}*\sum_{i=1}^{19} {YLDrate}^{Disease i}*{yld}_{SES}^{Disease i}$

The YLD burden for gender and socio-economic groups, ${YLD}_{SES}$, is distributed across age groups by using the relative contribution of each age group to overall YLD for gender, ${yld}_{Gender, Age}=\frac{{YLD}_{Age}}{{YLD}_{AllAges}}$.

1. ${YLD}_{SES, Age}={YLD}_{SES}*{yld}_{Age}$

YLD rates are then calculated using the population sizes from the 2018 Census ([Malawi], 2018).

1. ${YLDrate}_{SES, Age}=\frac{{YLD}_{SES, Age}}{{Population}_{SES,Age}}$

With the life expectancy and YLD estimates, we can calculate HALE for each socio-economic group using the life tables.

# Section 2: Supplement Tables

Table S1: Intervention Mapping for EHP interventions

| **Disease Area** | **Intervention** | **Survey / Source** | **Prevalence** | **Utilisation** | **Mean imputation?** |
| --- | --- | --- | --- | --- | --- |
| Environmental health | Child protection | DHS |  | Utilisation of fieldworkers, women recode V393 | Yes |
|  | Community Health Promotion & Engagement | DHS |  | Utilisation of fieldworkers, women recode V393 | Yes |
|  | Disease Surveillance | DHS |  | Utilisation of fieldworkers, women recode V393 | Yes |
|  | Growth Monitoring | DHS | Moderate malnutrition Prevalence, children recode HW8 / IHS hh_v08, hh_v09 | | Yes |
|  | Promotion of hygiene (Hand washing with soap) | DHS |  | Utilisation of fieldworkers, women recode V393 | Yes |
|  | Home-based care of chronically ill patients | DHS |  | Utilisation of fieldworkers, women recode V393 | Yes |
|  | Household water quality testing and treatment | DHS |  | Utilisation/Sources of drinking water, household recode HV201 / IHS hh_f36 | Yes |
|  | Occupational Health Promotion | DHS |  | Utilisation of fieldworkers, women recode V393 | Yes |
|  | Promotion of Sanitation (latrine refuse, drop hole covers, solid waste disposal, hygienic disposal of children’s stools) | DHS |  | Utilisation of fieldworkers, women recode V393 | Yes |
|  | Vermin and Vector Control & Promotion | DHS |  | Utilisation of fieldworkers, women recode V393 | Yes |
|  | Village Inspections | DHS |  | Utilisation of fieldworkers, women recode V393 | Yes |
| HIV & STIs | ART all ages | IHS | HIV Prevalence | IHS care seeking | No |
|  | Cotrimoxazole for children | IHS | HIV Prevalence in children |  | Yes |
|  | HIV Testing Services | IHS | HIV Prevalence | IHS care seeking | No |
|  | Paediatric ART | IHS | HIV Prevalence in children |  | Yes |
|  | PMTCT | IHS | HIV Prevalence in pregnant women | IHS care seeking | No |
|  | Viral load | IHS | HIV Prevalence | IHS care seeking | No |
| Malaria | Complicated (adults, injectable artesunate) | IHS | Malaria Prevalence | IHS care seeking | No |
|  | Complicated (children, injectable artesunate) | IHS | Malaria Prevalence | IHS care seeking | No |
|  | IPT (pregnant women) | MICS |  | Utilisation, wm recode MN13 | Yes |
|  | ITN distribution to pregnant women | | Prevalence of pregnant women |  | Yes |
|  | RDTs for under-5 | IHS | Malaria Prevalence | IHS care seeking | No |
|  | Uncomplicated - 2nd line (adult, <36 kg) | IHS | Malaria Prevalence | IHS care seeking | No |
|  | Uncomplicated - 2nd line (adult, >36 kg) | IHS | Malaria Prevalence | IHS care seeking | No |
|  | Uncomplicated - 2nd line (children, <15 kg) | IHS | Malaria Prevalence in U5 | IHS care seeking | No |
|  | Uncomplicated - 2nd line (children, >15 kg) | IHS | Malaria Prevalence in U5 | IHS care seeking | No |
|  | Uncomplicated (adult, <36 kg) | IHS | Malaria Prevalence | IHS care seeking | No |
|  | Uncomplicated (adult, >36 kg) | IHS | Malaria Prevalence | IHS care seeking | No |
|  | Uncomplicated (children, <15 kg) | IHS | Malaria Prevalence in U5 | IHS care seeking | No |
|  | Uncomplicated (children, >15 kg) | IHS | Malaria Prevalence in U5 | IHS care seeking | No |
| Mental Health | Anti-epileptic medication | IHS | Mental Illness Prevalence | IHS care seeking | No |
|  | Basic psychosocial support, advice, and follow-up | IHS | Mental Illness Prevalence | IHS care seeking | No |
|  | Treatment of depression | IHS | Mental Illness Prevalence | IHS care seeking | No |
| NCDs | Diabetes Type I | IHS | Diabetes Prevalence | IHS care seeking | No |
|  | Diabetes Type II | IHS | Diabetes Prevalence | IHS care seeking | No |
|  | Hypertension | IHS | Hypertension Prevalence | IHS care seeking | No |
|  | Testing of pre-cancerous cells (vinegar) | IHS | Cancer Prevalence | IHS care seeking | No |
|  | Treatment of Injuries (Blunt Trauma - Soft Tissue Injury) | IHS | Wounds Prevalence | IHS care seeking | No |
|  | Treatment of injuries (Fracture and dislocation) | IHS | Fractures Prevalence | IHS care seeking | No |
|  | Case finding and treatment of Trypanosomiasis | IHS | Schistosomiasis Prevalence | IHS care seeking | No |
|  | Schistosomiasis Mass drug administration | IHS | Schistosomiasis Prevalence | IHS care seeking | No |
|  | Trachoma mass drug administration | IHS | Eye Problem Prevalence | IHS care seeking | No |
| Nutrition | Community management of nutrition in under-5 - micronutrient powder | DHS | Moderate malnutrition Prevalence, children recode HW8 / IHS hh_v08, hh_v09 | | Yes |
|  | Community management of nutrition in under-5 - Plumpy Peanut | DHS | Moderate malnutrition Prevalence, children recode HW8 / IHS hh_v08, hh_v09 | | Yes |
|  | Community management of nutrition in under-5 - vitamin A | DHS | Moderate malnutrition Prevalence, children recode HW8 / IHS hh_v08, hh_v09 | | Yes |
|  | Deworming (children) | DHS | Severe malnutrition Prevalence, children recode HW7 / IHS hh_v08, hh_v09 | | Yes |
|  | Management of severe malnutrition (children) | DHS | Severe malnutrition Prevalence, children recode HW7 / IHS hh_v08, hh_v09 | | Yes |
|  | ORS | DHS | Moderate malnutrition Prevalence, children recode HW8 / IHS hh_v08, hh_v09 | Utilisation, children recode H13 | No |
|  | Vitamin A supplementation in infants and children 6-59 months | DHS | Moderate malnutrition Prevalence, children recode HW7 / IHS hh_v08, hh_v09 | | Yes |
|  | Vitamin A supplementation in pregnant women | IHS | Prevalence of pregnant women | IHS care seeking | No |
|  | Zinc | DHS | Moderate malnutrition Prevalence, children recode HW8 / IHS hh_v08, hh_v09 | Utilisation, children recode H15E | No |
| Oral Health | Management of mild tooth pain - tooth filling | IHS | Dental problems Prevalence | IHS care seeking | No |
|  | Management of severe tooth pain - tooth extraction | IHS | Dental problems Prevalence | IHS care seeking | No |
| RMNCH | Active management of the 3rd stage of labour | IHS | Prevalence of pregnant women | IHS care seeking | No |
|  | Antenatal corticosteroids for preterm labour | DHS | Prevalence of pregnant women | Utilisation, birth recode M17 | No |
|  | Antibiotics for pPRoM | DHS | Prevalence of pregnant women | Utilisation, birth recode M17 | No |
|  | Basic ANC | DHS | Prevalence of pregnant women | Utilisation, birth recode M14 | No |
|  | Caesarean section with indication | DHS | Prevalence of pregnant women | Utilisation, birth recode M17 | No |
|  | Caesarean Section with indication (with complication) | DHS | Prevalence of pregnant women | Utilisation, birth recode M17 | No |
|  | Clean practices and immediate essential new-born care (in facility) | IHS | Prevalence of pregnant women | Utilisation, birth recode M14 | No |
|  | Condoms | DHS |  | Utilisation, mother recode V307.5 | Yes |
|  | Cord care using chlorhexidine | IHS | Prevalence of pregnant women |  | Yes |
|  | Daily iron and folic acid supplementation (pregnant women) | IHS | Prevalence of pregnant women | IHS care seeking | No |
|  | Deworming (pregnant women) | IHS | Prevalence of pregnant women | IHS care seeking | No |
|  | Female sterilization | DHS |  | Utilisation, mothers recode V307.6 | Yes |
|  | Hysterectomy | DHS | Prevalence of Hysterectomy |  | Yes |
|  | Implant | DHS |  | Utilisation, mothers recode V307.11 | Yes |
|  | Injectable | DHS |  | Utilisation, mother recode V307.3 | Yes |
|  | IUD | DHS |  | Utilisation, mother recode V307.4 | Yes |
|  | Management of eclampsia | DHS | Prevalence of pregnant women | Utilisation, birth recode M17 | No |
|  | Management of obstructed labour | DHS | Prevalence of pregnant women | Utilisation, birth recode M17 | No |
|  | Management of pre-eclampsia | DHS | Prevalence of pregnant women | Utilisation, birth recode M17 | No |
|  | Maternal Sepsis case management | DHS | Prevalence of pregnant women | Utilisation, birth recode M17 | No |
|  | Neonatal resuscitation (institutional) | DHS | Prevalence of pregnant women | Utilisation, birth recode M17 | No |
|  | New-born sepsis - Full supportive care | DHS | Prevalence of pregnant women | Utilisation, birth recode M17 | No |
|  | New-born sepsis - Injectable antibiotics | DHS | Prevalence of pregnant women | Utilisation, birth recode M17 | No |
|  | Pill | DHS |  | Utilisation, mother recode V307.1 | Yes |
|  | Post-abortion case management | | |  |  |
|  | Syphilis detection and treatment (pregnant women) | IHS | Prevalence of pregnant women | Utilisation, birth recode M17 | No |
|  | Tetanus toxoid (pregnant women) | DHS, IHS | Prevalence of pregnant women | Utilisation, birth recode M1 | No |
|  | Treatment of antepartum haemorrhage | DHS |  | Utilisation, birth recode M17 | Yes |
|  | Treatment of postpartum haemorrhage | DHS |  | Utilisation, birth recode M17 | Yes |
|  | Treatment of severe diarrhoea | IHS | Prevalence of diarrhoea | IHS care seeking | No |
|  | Vaginal delivery - skilled attendance | DHS, IHS | Prevalence of pregnant women | Utilisation, birth recode M3b | No |
|  | Vaginal Delivery - with complication | DHS, IHS | Prevalence of pregnant women | Utilisation, birth recode M3b | No |
| TB | Case management of MDR cases | IHS | TB Prevalence | IHS care seeking | No |
|  | First line treatment for new TB Cases for adults | IHS | TB Prevalence | IHS care seeking | No |
|  | First line treatment for new TB Cases for children | IHS | TB Prevalence | IHS care seeking | No |
|  | First line treatment for retreatment TB Cases for adults | IHS | TB Prevalence | IHS care seeking | No |
|  | First line treatment for retreatment TB Cases for children | IHS | TB Prevalence | IHS care seeking | No |
|  | Isonized Preventive Therapy for children in contact with TB patients | IHS | TB Prevalence | IHS care seeking | No |
|  | LED test | IHS | TB Prevalence | IHS care seeking | No |
|  | LGA test | IHS | TB Prevalence | IHS care seeking | No |
|  | MGIT test | IHS | TB Prevalence | IHS care seeking | No |
|  | Xpert test | IHS | TB Prevalence | IHS care seeking | No |
|  | ZN test | IHS | TB Prevalence | IHS care seeking | No |
| Vaccine Preventable Diseases | BCG vaccine | DHS |  | Utilisation, birth recode H2 | Yes |
|  | DPT-Heb-Hib / Pentavalent vaccine | DHS |  | Utilisation, birth recode H51 | Yes |
|  | HPV vaccine | |  |  |  |
|  | Measles rubella vaccine | | |  |  |
|  | Pneumococcal vaccine | IHS | Pneumonia Prevalence | IHS care seeking | No |
|  | Pneumonia treatment (children) | IHS | Pneumonia Prevalence | IHS care seeking | No |
|  | Polio vaccine | DHS |  | Utilisation, birth recode H8 | Yes |
|  | Rotavirus vaccine | DHS |  | Utilisation, birth recode H57 | Yes |
|  | Treatment of severe pneumonia | IHS | Pneumonia Prevalence | IHS care seeking | No |

Table S2: Socio-economic distribution of mortality, mean (s.e.)

|  | | | | | | | | | | | | | | | | | | | | | | | |
| --- | --- | --- | --- | --- | --- | --- | --- | --- | --- | --- | --- | --- | --- | --- | --- | --- | --- | --- | --- | --- | --- | --- | --- |
| **Age-specific mortality rates for female population** | | | | | | | | | | | | | | | | | | | | | | | |
|  | **General** | | **Urban** | | | | **Rural** | | | **Poorest** | | | **Poorer** | | | **Middle** | | | **Richer** | | | **Richest** | |
| **Age** | **Deaths** | **Exposure** | | **Deaths** | **Exposure** | **Deaths** | | **Exposure** | **Deaths** | | **Exposure** | **Deaths** | | **Exposure** | **Deaths** | | **Exposure** | **Deaths** | | **Exposure** | **Deaths** | | **Exposure** |
| **Mortality in respondents’ children** | | | | | | | | | | | | | | | | | | | | | | | |
| 0-4 | 455 | 26096 | | 54 | 4016 | 401 | | 22080 | 103 | | 4801 | 119 | | 5711 | 108 | | 6203 | 68 | | 5024 | 57 | | 4357 |
| 5-9 | 69 | 18983 | | 8 | 3072 | 61 | | 15911 | 19 | | 3214 | 17 | | 3788 | 14 | | 4399 | 9 | | 4010 | 10 | | 3572 |
| 10-14 | 34 | 13179 | | 5 | 2146 | 29 | | 11033 | 5 | | 2100 | 8 | | 2513 | 11 | | 2931 | 9 | | 2934 | 1 | | 2701 |
| 15-19 | 23 | 7499 | | 3 | 1228 | 20 | | 6271 | 5 | | 1184 | 4 | | 1418 | 7 | | 1570 | 5 | | 1683 | 2 | | 1644 |
| 20-24 | 19 | 3617 | | 4 | 565 | 15 | | 3052 | 2 | | 585 | 6 | | 720 | 3 | | 758 | 4 | | 760 | 4 | | 794 |
| 25-29 | 8 | 1235 | | 0 | 173 | 8 | | 1062 | 2 | | 210 | 3 | | 246 | 0 | | 255 | 1 | | 251 | 2 | | 273 |
| 30-34 | 6 | 227 | | 0 | 32 | 6 | | 195 | 1 | | 30 | 3 | | 54 | 0 | | 50 | 2 | | 42 | 0 | | 51 |
| 35-39 | 1 | 9 | | 0 | 0 | 1 | | 9 | 0 | | 0 | 1 | | 3 | 0 | | 2 | 0 | | 1 | 0 | | 3 |
|  |  |  | |  |  |  | |  |  | |  |  | |  |  | |  |  | |  |  | |  |
| **Mortality in respondents’ siblings** | | | | | | | | | | | | | | | | | | | | | | | |
| 0-4 | 167 | 15969 | | 20 | 2478 | 147 | | 13491 | 52 | | 2977 | 50 | | 3614 | 31 | | 3502 | 26 | | 3130 | 8 | | 2746 |
| 5-9 | 59 | 16968 | | 1 | 3063 | 58 | | 13905 | 13 | | 2927 | 22 | | 3775 | 10 | | 3610 | 10 | | 3260 | 4 | | 3396 |
| 10-14 | 47 | 21827 | | 6 | 4275 | 41 | | 17552 | 12 | | 3654 | 10 | | 4674 | 7 | | 4521 | 10 | | 4193 | 8 | | 4785 |
| 15-19 | 65 | 25313 | | 17 | 5281 | 48 | | 20032 | 12 | | 4113 | 11 | | 5258 | 7 | | 5176 | 13 | | 4815 | 22 | | 5951 |
| 20-24 | 107 | 26052 | | 16 | 5674 | 91 | | 20378 | 18 | | 4111 | 26 | | 5141 | 16 | | 5253 | 20 | | 5040 | 27 | | 6507 |
| 24-29 | 132 | 23438 | | 37 | 5197 | 95 | | 18241 | 21 | | 3666 | 25 | | 4495 | 30 | | 4734 | 24 | | 4530 | 32 | | 6013 |
| 30-34 | 154 | 18114 | | 46 | 3970 | 108 | | 14144 | 26 | | 2757 | 24 | | 3341 | 31 | | 3749 | 28 | | 3596 | 45 | | 4671 |
| 35-39 | 147 | 12479 | | 35 | 2579 | 112 | | 9900 | 12 | | 1947 | 40 | | 2271 | 27 | | 2596 | 32 | | 2496 | 36 | | 3169 |
| 40-44 | 111 | 7526 | | 26 | 1478 | 85 | | 6048 | 14 | | 1114 | 23 | | 1373 | 14 | | 1582 | 31 | | 1554 | 29 | | 1903 |
| 45-49 | 66 | 4173 | | 9 | 791 | 57 | | 3382 | 9 | | 633 | 19 | | 768 | 14 | | 833 | 10 | | 844 | 14 | | 1095 |
| 50-54 | 56 | 2040 | | 14 | 398 | 42 | | 1642 | 13 | | 298 | 7 | | 373 | 13 | | 393 | 9 | | 422 | 14 | | 554 |
| 55-59 | 12 | 827 | | 4 | 169 | 8 | | 658 | 1 | | 132 | 0 | | 151 | 2 | | 143 | 3 | | 163 | 6 | | 238 |
| 60-64 | 9 | 308 | | 4 | 61 | 5 | | 247 | 0 | | 50 | 2 | | 67 | 2 | | 49 | 1 | | 57 | 4 | | 85 |
| 65-69 | 4 | 114 | | 3 | 20 | 1 | | 94 | 1 | | 15 | 0 | | 26 | 1 | | 26 | 0 | | 22 | 2 | | 25 |
| 70-74 | 1 | 30 | | 1 | 4 | 0 | | 26 | 0 | | 4 | 0 | | 10 | 0 | | 9 | 1 | | 4 | 0 | | 3 |
| 75-79 | 1 | 7 | | 0 | 1 | 1 | | 6 | 1 | | 2 | 0 | | 0 | 0 | | 3 | 0 | | 1 | 0 | | 1 |
| 80-84 | 0 | 2 | | 0 | 0 | 0 | | 2 | 0 | | 2 | 0 | | 0 | 0 | | 0 | 0 | | 0 | 0 | | 0 |
| 85-89 | 0 | 2 | | 0 | 0 | 0 | | 2 | 0 | | 2 | 0 | | 0 | 0 | | 0 | 0 | | 0 | 0 | | 0 |
| 90-94 | 0 | 1 | | 0 | 0 | 0 | | 1 | 0 | | 1 | 0 | | 0 | 0 | | 0 | 0 | | 0 | 0 | | 0 |
| 95+ | 0 | 0 | | 0 | 0 | 0 | | 0 | 0 | | 0 | 0 | | 0 | 0 | | 0 | 0 | | 0 | 0 | | 0 |
|  | | | | | | | | | | | | | | | | | | | | | | | |
| **Age-specific mortality rates for male population** | | | | | | | | | | | | | | | | | | | | | | | |
|  | | **General** | **Urban** | | | | **Rural** | | | **Poorest** | | | **Poorer** | | | **Middle** | | | **Richer** | | | **Richest** | |
| **Age** | **Deaths** | **Exposure** | | **Deaths** | **Exposure** | **Deaths** | | **Exposure** | **Deaths** | | **Exposure** | **Deaths** | | **Exposure** | **Deaths** | | **Exposure** | **Deaths** | | **Exposure** | **Deaths** | | **Exposure** |
| **Mortality in respondents’ children** | | | | | | | | | | | | | | | | | | | | | | | |
| 0-4 | 571 | 26433 | | 73 | 4170 | 498 | | 22263 | 120 | | 5005 | 156 | | 5673 | 105 | | 6162 | 114 | | 4992 | 76 | | 4601 |
| 5-9 | 70 | 18966 | | 12 | 3154 | 58 | | 15812 | 14 | | 3322 | 18 | | 3641 | 19 | | 4393 | 13 | | 3894 | 6 | | 3716 |
| 10-14 | 25 | 13168 | | 3 | 2211 | 22 | | 10957 | 9 | | 2133 | 1 | | 2370 | 3 | | 2984 | 3 | | 2945 | 9 | | 2736 |
| 15-19 | 27 | 7603 | | 2 | 1289 | 25 | | 6314 | 3 | | 1168 | 4 | | 1344 | 8 | | 1639 | 6 | | 1770 | 6 | | 1682 |
| 20-24 | 17 | 3469 | | 3 | 576 | 14 | | 2893 | 3 | | 501 | 2 | | 621 | 3 | | 740 | 6 | | 816 | 3 | | 791 |
| 25-29 | 9 | 1166 | | 2 | 197 | 7 | | 969 | 3 | | 178 | 2 | | 231 | 0 | | 233 | 3 | | 252 | 1 | | 272 |
| 30-34 | 4 | 216 | | 1 | 33 | 3 | | 183 | 0 | | 26 | 1 | | 48 | 1 | | 48 | 1 | | 50 | 1 | | 44 |
| 35-39 | 0 | 7 | | 0 | 0 | 0 | | 7 | 0 | | 1 | 0 | | 4 | 0 | | 0 | 0 | | 1 | 0 | | 1 |
|  |  |  | |  |  |  | |  |  | |  |  | |  |  | |  |  | |  |  | |  |
|  |  |  | |  |  |  | |  |  | |  |  | |  |  | |  |  | |  |  | |  |
| **Mortality in respondents’ siblings** | | | | | | | | | | | | | | | | | | | | | | | |
| 0-4 | 166 | 16724 | | 20 | 2669 | 146 | | 14055 | 48 | | 3075 | 43 | | 3730 | 31 | | 3768 | 33 | | 3143 | 11 | | 3008 |
| 5-9 | 62 | 16849 | | 15 | 3074 | 47 | | 13775 | 16 | | 2977 | 17 | | 3701 | 13 | | 3578 | 6 | | 3195 | 10 | | 3398 |
| 10-14 | 71 | 21287 | | 17 | 4210 | 54 | | 17077 | 17 | | 3594 | 13 | | 4409 | 16 | | 4466 | 11 | | 4137 | 14 | | 4681 |
| 15-19 | 82 | 24529 | | 17 | 5206 | 65 | | 19323 | 14 | | 4061 | 18 | | 4910 | 16 | | 5148 | 18 | | 4690 | 16 | | 5720 |
| 20-24 | 123 | 25416 | | 32 | 5565 | 91 | | 19851 | 29 | | 4099 | 19 | | 4905 | 23 | | 5280 | 22 | | 4959 | 30 | | 6173 |
| 24-29 | 136 | 23671 | | 33 | 5201 | 103 | | 18470 | 19 | | 3724 | 28 | | 4458 | 26 | | 4960 | 32 | | 4632 | 31 | | 5897 |
| 30-34 | 195 | 18757 | | 51 | 4012 | 144 | | 14745 | 25 | | 2899 | 42 | | 3419 | 42 | | 3892 | 42 | | 3757 | 44 | | 4790 |
| 35-39 | 186 | 13225 | | 44 | 2705 | 142 | | 10520 | 24 | | 1999 | 27 | | 2398 | 38 | | 2826 | 35 | | 2656 | 62 | | 3346 |
| 40-44 | 179 | 7784 | | 27 | 1453 | 152 | | 6331 | 28 | | 1207 | 30 | | 1450 | 36 | | 1646 | 43 | | 1585 | 42 | | 1896 |
| 45-49 | 102 | 4156 | | 21 | 744 | 81 | | 3412 | 24 | | 641 | 16 | | 797 | 20 | | 882 | 16 | | 878 | 26 | | 958 |
| 50-54 | 71 | 1910 | | 17 | 341 | 54 | | 1569 | 17 | | 306 | 10 | | 370 | 12 | | 355 | 13 | | 421 | 19 | | 458 |
| 55-59 | 35 | 805 | | 5 | 156 | 30 | | 649 | 7 | | 121 | 6 | | 151 | 5 | | 142 | 9 | | 173 | 8 | | 218 |
| 60-64 | 11 | 292 | | 1 | 60 | 10 | | 232 | 0 | | 42 | 2 | | 59 | 0 | | 44 | 3 | | 66 | 6 | | 81 |
| 65-69 | 7 | 103 | | 1 | 16 | 6 | | 87 | 0 | | 20 | 2 | | 19 | 1 | | 22 | 4 | | 24 | 0 | | 18 |
| 70-74 | 1 | 28 | | 0 | 4 | 1 | | 24 | 0 | | 5 | 0 | | 2 | 1 | | 7 | 0 | | 11 | 0 | | 3 |
| 75-79 | 1 | 12 | | 0 | 1 | 1 | | 11 | 0 | | 2 | 0 | | 2 | 0 | | 4 | 1 | | 4 | 0 | | 0 |
| 80-84 | 0 | 4 | | 0 | 0 | 0 | | 4 | 0 | | 0 | 0 | | 1 | 0 | | 2 | 0 | | 1 | 0 | | 0 |
| 85-89 | 0 | 2 | | 0 | 0 | 0 | | 2 | 0 | | 1 | 0 | | 1 | 0 | | 0 | 0 | | 0 | 0 | | 0 |
| 90-94 | 0 | 1 | | 0 | 0 | 0 | | 1 | 0 | | 1 | 0 | | 0 | 0 | | 0 | 0 | | 0 | 0 | | 0 |
| 95+ | 0 | 1 | | 0 | 0 | 0 | | 1 | 0 | | 1 | 0 | | 0 | 0 | | 0 | 0 | | 0 | 0 | | 0 |
|  |  |  | |  |  |  | |  |  | |  |  | |  |  | |  |  | |  |  | |  |

Table S3: YLD disease mapping

| **GBD disease** | **Total YLD in Malawi 2017** | **% of total YLD** | **Linked disease** | **Survey** |
| --- | --- | --- | --- | --- |
| Dietary iron deficiency | 133484 | 8.3 | Anaemia | DHS |
| HIV/AIDS | 100320 | 6.2 | HIV/AIDS | IHS |
| Low back pain | 95454 | 5.8 | Backache | IHS |
| Headache disorders | 79488 | 4.9 | Headache | IHS |
| Depressive disorders | 72157 | 4.5 | Mental health problems | IHS |
| Diabetes mellitus | 56346 | 3.5 | Diabetes | IHS |
| Anxiety disorders | 53159 | 3.3 | Mental health problems | IHS |
| Diarrheal diseases | 46485 | 2.9 | Diarrhoea | IHS |
| Dermatitis | 37267 | 2.3 | Skin problems | IHS |
| Blindness and vision impairment | 37089 | 2.3 | Eye problem | IHS |
| Epilepsy | 31872 | 2.0 | Epilepsy | IHS |
| Asthma | 28826 | 1.8 | Asthma | IHS |
| Malaria | 25385 | 1.5 | Malaria | IHS |
| Alcohol use disorders | 22480 | 1.4 | At least 1 drink almost every day in women | MICS |
| Oral disorders | 21371 | 1.3 | Dental problems | IHS |
| Bipolar disorder | 18000 | 1.1 | Mental health problems | IHS |
| Other mental disorders | 17657 | 1.1 | Mental health problems | IHS |
| Upper respiratory infection | 15036 | 0.8 | Upper respiratory infection | IHS |
| Schizophrenia | 11932 | 0.7 | Mental health problems | IHS |
| ALL causes | 1617535 | 100 |  |  |
|  |  |  |  |  |

Table S4: Intervention-specific population distribution, ranked by incremental net health benefit

| **Intervention** | **Incr. net benefit** | **Incr. DALY** | **Incr. Cost** | **EHP** | **Pop. in thousands** | **Eligible Population in thousands** | | | | | | | **Service Users in thousands** | | | | | | |
| --- | --- | --- | --- | --- | --- | --- | --- | --- | --- | --- | --- | --- | --- | --- | --- | --- | --- | --- | --- |
|  |  |  |  |  |  | **N (%)** | | | | | | | **N (%)** | | | | | | |
|  |  |  |  |  |  | **Urban** | **Rural** | **Poorest** | **Poorer** | **Middle** | **Richer** | **Richest** | **Urban** | **Rural** | **Poorest** | **Poorer** | **Middle** | **Richer** | **Richest** |
| Case management of MDR cases | 699.48 | 740.32 | 2491 | Y | 0 | 0 (16%) | 0 (84%) | 0 (40%) | 0 (12%) | 0 (24%) | 0 (11%) | 0 (14%) | 0 (49%) | 0 (46%) | 0 (37%) | 0 (49%) | 0 (50%) | 0 (64%) | 0 (50%) |
| Neonatal resuscitation (institutional) | 101.22 | 115.97 | 899 | Y | 5 | 1 (14%) | 5 (86%) | 2 (36%) | 1 (16%) | 1 (23%) | 1 (13%) | 1 (12%) | 0 (37%) | 2 (47%) | 1 (48%) | 0 (39%) | 1 (46%) | 0 (49%) | 0 (43%) |
| First line treatment for retreatment TB Cases for children | 42.09 | 72.26 | 1840 | Y | 39 | 6 (16%) | 33 (84%) | 16 (40%) | 5 (12%) | 9 (24%) | 4 (11%) | 5 (14%) | 3 (49%) | 15 (46%) | 6 (37%) | 2 (49%) | 5 (50%) | 3 (64%) | 3 (50%) |
| First line treatment for new TB Cases for adults | 42.09 | 72.26 | 1840 | Y | 39 | 6 (16%) | 33 (84%) | 16 (40%) | 5 (12%) | 9 (24%) | 4 (11%) | 5 (14%) | 3 (49%) | 15 (46%) | 6 (37%) | 2 (49%) | 5 (50%) | 3 (64%) | 3 (50%) |
| First line treatment for new TB Cases for children | 42.09 | 72.26 | 1840 | Y | 39 | 6 (16%) | 33 (84%) | 16 (40%) | 5 (12%) | 9 (24%) | 4 (11%) | 5 (14%) | 3 (49%) | 15 (46%) | 6 (37%) | 2 (49%) | 5 (50%) | 3 (64%) | 3 (50%) |
| First line treatment for retreatment TB Cases for adults | 42.09 | 72.26 | 1840 | Y | 39 | 6 (16%) | 33 (84%) | 16 (40%) | 5 (12%) | 9 (24%) | 4 (11%) | 5 (14%) | 3 (49%) | 15 (46%) | 6 (37%) | 2 (49%) | 5 (50%) | 3 (64%) | 3 (50%) |
| Management of pre-eclampsia | 27.25 | 30.20 | 180 | Y | 17 | 2 (14%) | 14 (86%) | 6 (36%) | 3 (16%) | 4 (23%) | 2 (13%) | 2 (12%) | 1 (37%) | 7 (47%) | 3 (48%) | 1 (39%) | 2 (46%) | 1 (49%) | 1 (43%) |
| Cesearian Section with indication (with complication) | 22.03 | 27.19 | 315 | Y | 1 | 0 (14%) | 1 (86%) | 0 (36%) | 0 (16%) | 0 (23%) | 0 (13%) | 0 (12%) | 0 (37%) | 0 (47%) | 0 (48%) | 0 (39%) | 0 (46%) | 0 (49%) | 0 (43%) |
| Management of obstructed labour | 22.03 | 27.19 | 315 | N | 77 | 11 (14%) | 66 (86%) | 28 (36%) | 12 (16%) | 18 (23%) | 10 (13%) | 9 (12%) | 4 (37%) | 31 (47%) | 13 (48%) | 5 (39%) | 8 (46%) | 5 (49%) | 4 (43%) |
| PMTCT | 8.07 | 9.78 | 104 | Y | 17 | 4 (25%) | 12 (75%) | 5 (32%) | 2 (12%) | 3 (18%) | 3 (19%) | 3 (19%) | 1 (18%) | 3 (25%) | 1 (29%) | 0 (23%) | 1 (19%) | 1 (21%) | 1 (20%) |
| Induction of labour (beyond 41 weeks) | 6.91 | 7.31 | 24 | Y | 38 | 5 (14%) | 33 (86%) | 14 (36%) | 6 (16%) | 9 (23%) | 5 (13%) | 5 (12%) | 2 (37%) | 15 (47%) | 7 (48%) | 2 (39%) | 4 (46%) | 2 (49%) | 2 (43%) |
| Active management of the 3rd stage of labour | 6.90 | 7.30 | 24 | N | 765 | 91 (12%) | 675 (88%) | 231 (30%) | 140 (18%) | 221 (29%) | 134 (18%) | 39 (5%) | 63 (70%) | 492 (73%) | 102 (44%) | 130 (93%) | 205 (93%) | 118 (88%) | 0 (1%) |
| Management of eclampsia | 6.86 | 7.29 | 26 | Y | 14 | 2 (14%) | 12 (86%) | 5 (36%) | 2 (16%) | 3 (23%) | 2 (13%) | 2 (12%) | 1 (37%) | 6 (47%) | 2 (48%) | 1 (39%) | 1 (46%) | 1 (49%) | 1 (43%) |
| Male circumcision | 6.22 | 9.73 | 214 | Y | 259 | 36 (14%) | 223 (86%) | 94 (36%) | 40 (16%) | 60 (23%) | 34 (13%) | 31 (12%) | 13 (37%) | 104 (47%) | 45 (48%) | 16 (39%) | 28 (46%) | 17 (49%) | 13 (43%) |
| Cesearian section with indication | 4.58 | 9.64 | 309 | N | 4 | 1 (14%) | 3 (86%) | 1 (36%) | 1 (16%) | 1 (23%) | 1 (13%) | 0 (12%) | 0 (37%) | 2 (47%) | 1 (48%) | 0 (39%) | 0 (46%) | 0 (49%) | 0 (43%) |
| Pneumonia treatment (children) | 4.30 | 4.71 | 25 | N | 79 | 8 (10%) | 71 (90%) | 31 (40%) | 16 (21%) | 16 (20%) | 8 (10%) | 8 (10%) | 1 (18%) | 14 (20%) | 7 (22%) | 6 (34%) | 2 (11%) | 1 (9%) | 1 (9%) |
| Kangaroo mother care | 1.43 | 1.83 | 24 | N | 99 | 12 (12%) | 88 (88%) | 30 (30%) | 18 (18%) | 29 (29%) | 17 (18%) | 5 (5%) | 8 (70%) | 64 (73%) | 13 (44%) | 17 (93%) | 27 (93%) | 15 (88%) | 0 (1%) |
| Interventions focused on men who have sex with men | 1.14 | 6.87 | 349 | N | 7 | 1 (14%) | 9 (86%) | 4 (36%) | 2 (16%) | 2 (23%) | 1 (13%) | 1 (12%) | 1 (37%) | 4 (47%) | 2 (48%) | 1 (39%) | 1 (46%) | 1 (49%) | 1 (43%) |
| Interventions focused on female sex workers | 1.14 | 6.87 | 349 | N | 10 | 1 (14%) | 6 (86%) | 2 (36%) | 1 (16%) | 2 (23%) | 1 (13%) | 1 (12%) | 0 (37%) | 3 (47%) | 1 (48%) | 0 (39%) | 1 (46%) | 0 (49%) | 0 (43%) |
| First trimester - uncomplicated | 1.01 | 1.10 | 6 | Y | 191 | 27 (14%) | 164 (86%) | 64 (34%) | 28 (14%) | 49 (26%) | 26 (13%) | 25 (13%) | 14 (52%) | 96 (58%) | 38 (60%) | 15 (56%) | 29 (59%) | 15 (57%) | 13 (52%) |
| Second trimester - uncomplicated | 1.01 | 1.10 | 6 | Y | 191 | 23 (12%) | 169 (88%) | 58 (30%) | 35 (18%) | 55 (29%) | 34 (18%) | 10 (5%) | 16 (70%) | 123 (73%) | 25 (44%) | 33 (93%) | 51 (93%) | 29 (88%) | 0 (1%) |
| Uncomplicated - 2nd line (children, <15 kg) | 0.92 | 0.99 | 4 | Y | 325 | 39 (12%) | 286 (88%) | 127 (39%) | 60 (18%) | 86 (27%) | 27 (8%) | 24 (7%) | 24 (61%) | 191 (67%) | 87 (68%) | 38 (63%) | 57 (66%) | 18 (68%) | 14 (60%) |
| Uncomplicated - 2nd line (children, >15 kg) | 0.92 | 0.99 | 4 | Y | 325 | 39 (12%) | 286 (88%) | 127 (39%) | 60 (18%) | 86 (27%) | 27 (8%) | 24 (7%) | 24 (61%) | 191 (67%) | 87 (68%) | 38 (63%) | 57 (66%) | 18 (68%) | 14 (60%) |
| Uncomplicated (children, >15 kg) | 0.92 | 0.99 | 4 | Y | 325 | 39 (12%) | 286 (88%) | 127 (39%) | 60 (18%) | 86 (27%) | 27 (8%) | 24 (7%) | 24 (61%) | 191 (67%) | 87 (68%) | 38 (63%) | 57 (66%) | 18 (68%) | 14 (60%) |
| Uncomplicated (children, <15 kg) | 0.92 | 0.99 | 4 | Y | 325 | 39 (12%) | 286 (88%) | 127 (39%) | 60 (18%) | 86 (27%) | 27 (8%) | 24 (7%) | 24 (61%) | 191 (67%) | 87 (68%) | 38 (63%) | 57 (66%) | 18 (68%) | 14 (60%) |
| Management of severe malnutrition (children) | 0.69 | 3.91 | 196 | Y | 33 | 5 (14%) | 28 (86%) | 12 (36%) | 5 (16%) | 8 (23%) | 4 (13%) | 4 (12%) | 2 (37%) | 13 (47%) | 6 (48%) | 2 (39%) | 3 (46%) | 2 (49%) | 2 (43%) |
| Syphilis detection and treatment (pregnant women) | 0.57 | 1.00 | 26 | N | 765 | 91 (12%) | 675 (88%) | 231 (30%) | 140 (18%) | 221 (29%) | 134 (18%) | 39 (5%) | 63 (70%) | 492 (73%) | 102 (44%) | 130 (93%) | 205 (93%) | 118 (88%) | 0 (1%) |
| Newborn sepsis - Full supportive care | 0.53 | 0.87 | 21 | N | 64 | 9 (14%) | 55 (86%) | 23 (36%) | 10 (16%) | 15 (23%) | 8 (13%) | 8 (12%) | 3 (37%) | 26 (47%) | 11 (48%) | 4 (39%) | 7 (46%) | 4 (49%) | 3 (43%) |
| Pregnant Women - complicated | 0.49 | 0.53 | 3 | N | 13 | 2 (12%) | 11 (88%) | 4 (30%) | 2 (18%) | 4 (29%) | 2 (18%) | 1 (5%) | 1 (70%) | 8 (73%) | 2 (44%) | 2 (93%) | 3 (93%) | 2 (88%) | 0 (1%) |
| Management of moderate acute malnutrition (children) | 0.47 | 1.17 | 43 | N | 68 | 8 (12%) | 61 (88%) | 21 (30%) | 13 (18%) | 20 (29%) | 12 (18%) | 3 (5%) | 6 (70%) | 44 (73%) | 9 (44%) | 12 (93%) | 18 (93%) | 11 (88%) | 0 (1%) |
| Management of moderate acute malnutrition (children) | 0.47 | 1.17 | 43 | Y | 68 | 9 (14%) | 59 (86%) | 25 (36%) | 11 (16%) | 16 (23%) | 9 (13%) | 8 (12%) | 4 (37%) | 27 (47%) | 12 (48%) | 4 (39%) | 7 (46%) | 4 (49%) | 3 (43%) |
| Management of moderate acute malnutrition (pregnant and lactating women) | 0.47 | 1.17 | 43 | Y | 69 | 9 (14%) | 59 (86%) | 25 (36%) | 11 (16%) | 16 (23%) | 9 (13%) | 8 (12%) | 4 (37%) | 27 (47%) | 12 (48%) | 4 (39%) | 7 (46%) | 4 (49%) | 3 (43%) |
| ITN distribution to pregnant women | 0.29 | 0.36 | 5 | Y | 765 | 109 (14%) | 656 (86%) | 257 (34%) | 111 (14%) | 197 (26%) | 102 (13%) | 98 (13%) | 57 (52%) | 383 (58%) | 153 (60%) | 61 (56%) | 116 (59%) | 58 (57%) | 51 (52%) |
| Vitamin A supplementation in pregnant women | 0.27 | 0.30 | 2 | Y | 103 | 12 (12%) | 91 (88%) | 31 (30%) | 19 (18%) | 30 (29%) | 18 (18%) | 5 (5%) | 9 (70%) | 66 (73%) | 14 (44%) | 18 (93%) | 28 (93%) | 16 (88%) | 0 (1%) |
| Anti-epileptic medication | 0.25 | 0.46 | 13 | Y | 485 | 59 (12%) | 426 (88%) | 249 (51%) | 58 (12%) | 83 (17%) | 51 (10%) | 45 (9%) | 5 (8%) | 82 (19%) | 33 (13%) | 14 (24%) | 25 (30%) | 6 (12%) | 9 (21%) |
| Antenatal corticosteroids for preterm labour | 0.19 | 0.32 | 8 | Y | 138 | 19 (14%) | 119 (86%) | 50 (36%) | 21 (16%) | 32 (23%) | 18 (13%) | 16 (12%) | 7 (37%) | 55 (47%) | 24 (48%) | 8 (39%) | 15 (46%) | 9 (49%) | 7 (43%) |
| Antibiotics for pPRoM | 0.18 | 0.52 | 21 | Y | 54 | 7 (14%) | 46 (86%) | 19 (36%) | 8 (16%) | 12 (23%) | 7 (13%) | 6 (12%) | 3 (37%) | 22 (47%) | 9 (48%) | 3 (39%) | 6 (46%) | 3 (49%) | 3 (43%) |
| Cotrimoxazole for children | 0.18 | 0.00 | -11 | Y | 106 | 7 (7%) | 99 (93%) | 33 (31%) | 15 (14%) | 29 (27%) | 0 (0%) | 29 (28%) | 0 (0%) | 20 (21%) | 8 (25%) | 0 (0%) | 0 (0%) | 0 (100%) | 12 (41%) |
| Maternal Sepsis case management | 0.13 | 0.35 | 14 | Y | 54 | 7 (14%) | 46 (86%) | 19 (36%) | 8 (16%) | 12 (23%) | 7 (13%) | 6 (12%) | 3 (37%) | 22 (47%) | 9 (48%) | 3 (39%) | 6 (46%) | 3 (49%) | 3 (43%) |
| Rotavirus vaccine | 0.12 | 0.14 | 1 | Y | 521 | 72 (14%) | 449 (86%) | 188 (36%) | 81 (16%) | 122 (23%) | 69 (13%) | 61 (12%) | 27 (37%) | 210 (47%) | 90 (48%) | 31 (39%) | 55 (46%) | 34 (49%) | 27 (43%) |
| Tetanus toxoid (pregnant women) | 0.11 | 0.13 | 1 | Y | 765 | 106 (14%) | 659 (86%) | 276 (36%) | 119 (16%) | 178 (23%) | 101 (13%) | 90 (12%) | 40 (37%) | 308 (47%) | 132 (48%) | 46 (39%) | 81 (46%) | 50 (49%) | 39 (43%) |
| Uncomplicated (adult, <36 kg) | 0.11 | 0.12 | 0 | Y | 2282 | 325 (14%) | 1957 (86%) | 765 (34%) | 330 (14%) | 588 (26%) | 306 (13%) | 293 (13%) | 170 (52%) | 1143 (58%) | 458 (60%) | 183 (56%) | 346 (59%) | 173 (57%) | 153 (52%) |
| Uncomplicated (adult, >36 kg) | 0.11 | 0.12 | 0 | Y | 2282 | 325 (14%) | 1957 (86%) | 765 (34%) | 330 (14%) | 588 (26%) | 306 (13%) | 293 (13%) | 170 (52%) | 1143 (58%) | 458 (60%) | 183 (56%) | 346 (59%) | 173 (57%) | 153 (52%) |
| Uncomplicated - 2nd line (adult, <36 kg) | 0.11 | 0.12 | 0 | Y | 2282 | 325 (14%) | 1957 (86%) | 765 (34%) | 330 (14%) | 588 (26%) | 306 (13%) | 293 (13%) | 170 (52%) | 1143 (58%) | 458 (60%) | 183 (56%) | 346 (59%) | 173 (57%) | 153 (52%) |
| Uncomplicated - 2nd line (adult, >36 kg) | 0.11 | 0.12 | 0 | Y | 2282 | 325 (14%) | 1957 (86%) | 765 (34%) | 330 (14%) | 588 (26%) | 306 (13%) | 293 (13%) | 170 (52%) | 1143 (58%) | 458 (60%) | 183 (56%) | 346 (59%) | 173 (57%) | 153 (52%) |
| Basic ANC | 0.08 | 0.11 | 2 | Y | 765 | 106 (14%) | 659 (86%) | 276 (36%) | 119 (16%) | 178 (23%) | 101 (13%) | 90 (12%) | 40 (37%) | 308 (47%) | 132 (48%) | 46 (39%) | 81 (46%) | 50 (49%) | 39 (43%) |
| Vaginal delivery - skilled attendance | 0.08 | 0.10 | 1 | Y | 647 | 90 (14%) | 557 (86%) | 234 (36%) | 100 (16%) | 151 (23%) | 85 (13%) | 76 (12%) | 34 (37%) | 260 (47%) | 111 (48%) | 39 (39%) | 69 (46%) | 42 (49%) | 33 (43%) |
| Vaginal Delivery - with complication | 0.08 | 0.10 | 1 | Y | 114 | 16 (14%) | 98 (86%) | 41 (36%) | 18 (16%) | 27 (23%) | 15 (13%) | 13 (12%) | 6 (37%) | 46 (47%) | 20 (48%) | 7 (39%) | 12 (46%) | 7 (49%) | 6 (43%) |
| HIV Testing Services | 0.04 | 0.06 | 2 | N | 3513 | 887 (25%) | 2625 (75%) | 1110 (32%) | 431 (12%) | 628 (18%) | 672 (19%) | 672 (19%) | 158 (18%) | 650 (25%) | 317 (29%) | 97 (23%) | 119 (19%) | 140 (21%) | 135 (20%) |
| Schistosomiasis Mass drug administration | 0.03 | 0.06 | 2 | N | 6217 | 279 (4%) | 5938 (96%) | 2638 (42%) | 1248 (20%) | 1474 (24%) | 659 (11%) | 197 (3%) | 0 (0%) | 2026 (34%) | 1271 (48%) | 0 (0%) | 262 (18%) | 296 (45%) | 197 (100%) |
| Ischemic heart disease | 0.00 | 0.00 | 0 | N | 135 | 25 (18%) | 110 (82%) | 52 (38%) | 21 (16%) | 21 (15%) | 17 (13%) | 24 (18%) | 16 (64%) | 74 (67%) | 35 (68%) | 18 (86%) | 9 (42%) | 13 (77%) | 14 (57%) |
| High Cholesterol | 0.00 | 0.00 | 0 | Y | 240 | 44 (18%) | 196 (82%) | 92 (38%) | 37 (16%) | 37 (15%) | 31 (13%) | 43 (18%) | 28 (64%) | 131 (67%) | 63 (68%) | 32 (86%) | 15 (42%) | 24 (77%) | 25 (57%) |
| GIT, Intestine cancer | 0.00 | 0.00 | 0 | N | 0 | 0 (7%) | 0 (93%) | 0 (15%) | 0 (27%) | 0 (27%) | 0 (24%) | 0 (7%) | 0 (0%) | 0 (71%) | 0 (69%) | 0 (100%) | 0 (17%) | 0 (100%) | 0 (0%) |
| Treatment of depression | 0.00 | 0.00 | 0 | Y | 866 | 106 (12%) | 760 (88%) | 445 (51%) | 103 (12%) | 148 (17%) | 91 (10%) | 80 (9%) | 8 (8%) | 147 (19%) | 59 (13%) | 25 (24%) | 44 (30%) | 11 (12%) | 16 (21%) |
| Treatment of bipolar disorder | 0.00 | 0.00 | 0 | N | 173 | 21 (12%) | 152 (88%) | 89 (51%) | 21 (12%) | 30 (17%) | 18 (10%) | 16 (9%) | 2 (8%) | 29 (19%) | 12 (13%) | 5 (24%) | 9 (30%) | 2 (12%) | 3 (21%) |
| IPT (pregnant women) | 0.00 | 0.00 | 0 | N | 765 | 106 (14%) | 659 (86%) | 276 (36%) | 119 (16%) | 178 (23%) | 101 (13%) | 90 (12%) | 40 (37%) | 308 (47%) | 132 (48%) | 46 (39%) | 81 (46%) | 50 (49%) | 39 (43%) |
| Treatment of schizophrenia | 0.00 | 0.00 | 0 | Y | 35 | 4 (12%) | 30 (88%) | 18 (51%) | 4 (12%) | 6 (17%) | 4 (10%) | 3 (9%) | 0 (8%) | 6 (19%) | 2 (13%) | 1 (24%) | 2 (30%) | 0 (12%) | 1 (21%) |
| Treatment of acute psychotic disorders | 0.00 | 0.00 | 0 | Y | 173 | 21 (12%) | 152 (88%) | 89 (51%) | 21 (12%) | 30 (17%) | 18 (10%) | 16 (9%) | 2 (8%) | 29 (19%) | 12 (13%) | 5 (24%) | 9 (30%) | 2 (12%) | 3 (21%) |
| Diabetes Type I | 0.00 | 0.00 | 0 | N | 24 | 14 (57%) | 10 (43%) | 2 (9%) | 1 (3%) | 1 (4%) | 4 (15%) | 17 (69%) | 2 (15%) | 3 (33%) | 1 (52%) | 0 (25%) | 0 (45%) | 0 (13%) | 3 (19%) |
| Diabetes Type II | 0.00 | 0.00 | 0 | Y | 151 | 87 (57%) | 64 (43%) | 14 (9%) | 4 (3%) | 6 (4%) | 22 (15%) | 104 (69%) | 13 (15%) | 21 (33%) | 7 (52%) | 1 (25%) | 3 (45%) | 3 (13%) | 20 (19%) |
| Cervical cancer (first line) | -0.01 | 0.00 | 0 | Y | 2 | 0 (7%) | 2 (93%) | 0 (15%) | 0 (27%) | 0 (27%) | 0 (24%) | 0 (7%) | 0 (0%) | 1 (71%) | 0 (69%) | 0 (100%) | 0 (17%) | 0 (100%) | 0 (0%) |
| ORS | -0.02 | 0.02 | 3 | Y | 6393 | 886 (14%) | 5507 (86%) | 2310 (36%) | 994 (16%) | 1491 (23%) | 845 (13%) | 754 (12%) | 332 (37%) | 2572 (47%) | 1101 (48%) | 383 (39%) | 679 (46%) | 414 (49%) | 327 (43%) |
| Zinc | -0.02 | 0.03 | 3 | N | 6297 | 873 (14%) | 5424 (86%) | 2275 (36%) | 979 (16%) | 1469 (23%) | 832 (13%) | 743 (12%) | 327 (37%) | 2533 (47%) | 1084 (48%) | 377 (39%) | 669 (46%) | 408 (49%) | 322 (43%) |
| Hypertension | -0.09 | 0.05 | 8 | Y | 887 | 250 (28%) | 637 (72%) | 202 (23%) | 64 (7%) | 155 (17%) | 119 (13%) | 347 (39%) | 118 (47%) | 425 (67%) | 162 (80%) | 45 (70%) | 42 (27%) | 85 (72%) | 209 (60%) |
| COPD | -0.11 | 0.07 | 11 | N | 443 | 103 (23%) | 340 (77%) | 150 (34%) | 46 (10%) | 97 (22%) | 53 (12%) | 96 (22%) | 29 (28%) | 110 (32%) | 50 (33%) | 17 (36%) | 26 (26%) | 19 (35%) | 28 (29%) |
| Daily iron and folic acid supplementation (pregnant women) | -0.21 | 0.19 | 24 | Y | 237 | 28 (12%) | 209 (88%) | 72 (30%) | 43 (18%) | 68 (29%) | 42 (18%) | 12 (5%) | 20 (70%) | 153 (73%) | 32 (44%) | 40 (93%) | 63 (93%) | 37 (88%) | 0 (1%) |
| Hypertensive disorder case management | -0.31 | 0.10 | 25 | N | 9 | 3 (28%) | 6 (72%) | 2 (23%) | 1 (7%) | 2 (17%) | 1 (13%) | 4 (39%) | 1 (47%) | 4 (67%) | 2 (80%) | 0 (70%) | 0 (27%) | 1 (72%) | 2 (60%) |
| Post-abortion case management | -0.46 | 0.05 | 31 | Y | 62 | 9 (14%) | 54 (86%) | 23 (36%) | 10 (16%) | 15 (23%) | 8 (13%) | 7 (12%) | 3 (37%) | 25 (47%) | 11 (48%) | 4 (39%) | 7 (46%) | 4 (49%) | 3 (43%) |
| Ectopic case management | -0.46 | 0.02 | 29 | N | 18 | 3 (14%) | 16 (86%) | 7 (36%) | 3 (16%) | 4 (23%) | 2 (13%) | 2 (12%) | 1 (37%) | 7 (47%) | 3 (48%) | 1 (39%) | 2 (46%) | 1 (49%) | 1 (43%) |
| BCG vaccine | -0.91 | 0.02 | 57 | Y | 521 | 72 (14%) | 449 (86%) | 188 (36%) | 81 (16%) | 122 (23%) | 69 (13%) | 61 (12%) | 27 (37%) | 210 (47%) | 90 (48%) | 31 (39%) | 55 (46%) | 34 (49%) | 27 (43%) |
| Antibiotics for treatment of dysentery | -1.03 | 0.30 | 81 | Y | 315 | 50 (16%) | 265 (84%) | 118 (37%) | 52 (17%) | 74 (24%) | 32 (10%) | 39 (12%) | 25 (51%) | 151 (57%) | 71 (60%) | 31 (60%) | 39 (53%) | 20 (62%) | 15 (39%) |
| Polio vaccine | -1.97 | 0.02 | 122 | Y | 521 | 72 (14%) | 449 (86%) | 188 (36%) | 81 (16%) | 122 (23%) | 69 (13%) | 61 (12%) | 27 (37%) | 210 (47%) | 90 (48%) | 31 (39%) | 55 (46%) | 34 (49%) | 27 (43%) |
| Pneumococcal vaccine | -6.19 | 1.54 | 471 | Y | 521 | 51 (10%) | 470 (90%) | 206 (40%) | 108 (21%) | 102 (20%) | 50 (10%) | 55 (10%) | 9 (18%) | 93 (20%) | 45 (22%) | 36 (34%) | 12 (11%) | 5 (9%) | 5 (9%) |
|  |  |  |  |  |  |  |  |  |  |  |  |  |  |  |  |  |  |  |  |
| Average DALY averted per EHP service use: | | | | | | | | | | | | | 0.73 | 0.81 | 1.18 | 0.67 | 1.14 | 0.65 | 0.34 |
| Average incremental cost per EHP service use: | | | | | | | | | | | | | $16.37 | $18.18 | $30.98 | $15.40 | $21.07 | $12.40 | $9.73 |
|  | | | | | | | | | | | | |  |  |  |  |  |  |  |
|  |  |  |  |  |  |  |  |  |  |  |  |  |  |  |  |  |  |  |  |
| Average DALY per not used EHP service: | | | | | | | | | | | | | 0.73 | 0.74 | 1.64 | 0.47 | 0.76 | 0.34 | 0.49 |
| Average incremental cost per not used EHP service: | | | | | | | | | | | | | $25.19 | $28.43 | $56.91 | $20.90 | $30.53 | $14.07 | $17.37 |
|  |  |  |  |  |  |  |  |  |  |  |  |  |  |  |  |  |  |  |  |

Table S5: Net population impact and distributional impact per intervention, ranked by EDE improvements (ΔEDE)

| **Disease Area** | **Intervention** | **Rank ΔEDE** | **ΔEDE** | **Rank CE** | **NPB** | **ΔEDE - NPB** | **Equity Plane Quadrant** |
| --- | --- | --- | --- | --- | --- | --- | --- |
| RMNCH | Active management of the 3rd stage of labour | 1 | 3,374,628 | 12 | 3,830,452 | -455,824 | +- |
| RMNCH | Management of obstructed labour | 2 | 877,074 | 10 | 765,731 | 111,343 | ++ |
| HIV & STIs | Male circumcision | 3 | 863,649 | 14 | 732,511 | 131,138 | ++ |
| TB | First line treatment for retreatment TB Cases for children | 4 | 861,355 | 6 | 763,706 | 97,649 | ++ |
| TB | First line treatment for retreatment TB Cases for adults | 5 | 861,355 | 5 | 763,706 | 97,649 | ++ |
| TB | First line treatment for new TB Cases for adults | 6 | 861,355 | 3 | 763,706 | 97,649 | ++ |
| TB | First line treatment for new TB Cases for children | 7 | 861,355 | 4 | 763,706 | 97,649 | ++ |
| RMNCH | Neonatal resuscitation (institutional) | 8 | 279,029 | 2 | 245,260 | 33,769 | ++ |
| RMNCH | Syphilis detection and treatment (pregnant women) | 9 | 252,831 | 27 | 316,109 | -63,278 | +- |
| RMNCH | Management of pre-eclampsia | 10 | 234,181 | 7 | 206,529 | 27,652 | ++ |
| Malaria | Uncomplicated - 2nd line (children, >15 kg) | 11 | 222,333 | 23 | 198,222 | 24,111 | ++ |
| Malaria | Uncomplicated - 2nd line (children, <15 kg) | 12 | 222,333 | 22 | 198,222 | 24,111 | ++ |
| Malaria | Uncomplicated (children, >15 kg) | 13 | 222,333 | 24 | 198,222 | 24,111 | ++ |
| Malaria | Uncomplicated (children, <15 kg) | 14 | 222,333 | 25 | 198,222 | 24,111 | ++ |
| Malaria | Uncomplicated (adult, <36 kg) | 15 | 159,180 | 42 | 145,458 | 13,722 | ++ |
| Malaria | Uncomplicated - 2nd line (adult, <36 kg) | 16 | 159,180 | 44 | 145,458 | 13,722 | ++ |
| Malaria | Uncomplicated (adult, >36 kg) | 17 | 159,180 | 43 | 145,458 | 13,722 | ++ |
| Malaria | Uncomplicated - 2nd line (adult, >36 kg) | 18 | 159,180 | 45 | 145,458 | 13,722 | ++ |
| Malaria | ITN distribution to pregnant women | 19 | 139,460 | 33 | 125,780 | 13,680 | ++ |
| RMNCH | Induction of labour (beyond 41 weeks) | 20 | 135,586 | 11 | 120,113 | 15,473 | ++ |
| Malaria | Second trimester - uncomplicated | 21 | 123,690 | 21 | 140,040 | -16,351 | +- |
| Malaria | First trimester - uncomplicated | 22 | 121,718 | 20 | 111,053 | 10,665 | ++ |
| NTDs | Schistosomiasis Mass drug administration | 23 | 106,582 | 50 | 63,756 | 42,826 | ++ |
| RMNCH | Kangaroo mother care | 24 | 88,819 | 17 | 103,286 | -14,467 | +- |
| IMCI | Pneumonia treatment (children) | 25 | 77,398 | 16 | 66,449 | 10,950 | ++ |
| RMNCH | Management of eclampsia | 26 | 48,456 | 13 | 42,897 | 5,558 | ++ |
| RMNCH | Tetanus toxoid (pregnant women) | 27 | 44,815 | 41 | 39,452 | 5,363 | ++ |
| HIV & STIs | HIV Testing Services | 28 | 38,423 | 49 | 29,908 | 8,515 | ++ |
| HIV & STIs | PMTCT | 28 | 37,125 | 10 | 30,751 | 6,374 | ++ |
| TB | Case management of MDR cases | 29 | 34,548 | 1 | 31,783 | 2,766 | ++ |
| RMNCH | Basic ANC | 31 | 33,831 | 46 | 29,262 | 4,570 | ++ |
| Vaccine Preventable Diseases | Rotavirus vaccine | 32 | 33,395 | 40 | 29,488 | 3,907 | ++ |
| RMNCH | Vaginal delivery - skilled attendance | 33 | 25,783 | 47 | 22,443 | 3,339 | ++ |
| Mental Health | Anti-epileptic medication | 34 | 24,947 | 35 | 21,276 | 3,671 | ++ |
| Nutrition | Management of moderate acute malnutrition (children) | 35 | 18,708 | 30 | 14,643 | 4,065 | ++ |
| Nutrition | Management of moderate acute malnutrition (children) | 36 | 18,708 | 31 | 14,643 | 4,065 | ++ |
| RMNCH | Newborn sepsis - Full supportive care | 37 | 18,246 | 28 | 15,317 | 2,929 | ++ |
| Nutrition | Vitamin A supplementation in pregnant women | 38 | 17,709 | 34 | 20,166 | -2,457 | +- |
| Nutrition | Management of moderate acute malnutrition (pregnant and lactating women) | 39 | 16,820 | 32 | 23,618 | -6,798 | +- |
| Nutrition | Management of severe malnutrition (children) | 40 | 16,556 | 26 | 10,286 | 6,270 | ++ |
| RMNCH | Antenatal corticosteroids for preterm labour | 41 | 14,270 | 36 | 11,941 | 2,329 | ++ |
| RMNCH | Cesearian section with indication | 42 | 9,865 | 15 | 7,964 | 1,901 | ++ |
| HIV & STIs | Interventions focused on female sex workers | 43 | 8,799 | 18 | 5,334 | 3,465 | ++ |
| RMNCH | Cesearian Section with indication (with complication) | 44 | 8,787 | 8 | 7,657 | 1,130 | ++ |
| HIV & STIs | Interventions focused on men who have sex with men | 45 | 5,883 | 19 | 3,566 | 2,317 | ++ |
| RMNCH | Antibiotics for pPRoM | 46 | 5,842 | 37 | 4,437 | 1,405 | ++ |
| RMNCH | Vaginal Delivery - with complication | 47 | 4,546 | 48 | 3,957 | 589 | ++ |
| Malaria | Pregnant Women - complicated | 48 | 4,068 | 29 | 4,605 | -537 | +- |
| RMNCH | Maternal Sepsis case management | 49 | 4,061 | 39 | 3,105 | 956 | ++ |
| HIV & STIs | Cotrimoxazole for children | 50 | 3,721 | 38 | 3,638 | 82 | ++ |
| NCDs | High Cholesterol | 51 | 29 | 52 | -74 | 102 | -+ |
| NCDs | GIT, Intestine cancer | 52 | 0 | 53 | 0 | 0 | -- |
| NCDs | Cervical cancer (first line) | 53 | -7 | 61 | -7 | 0 | -- |
| NCDs | Diabetes Type I | 54 | -22 | 59 | -23 | 0 | -+ |
| Mental Health | Treatment of schizophrenia | 55 | -26 | 58 | -26 | 0 | -- |
| NCDs | Ischemic heart disease | 56 | -31 | 51 | -32 | 0 | -+ |
| Mental Health | Treatment of bipolar disorder | 57 | -88 | 55 | -87 | -1 | -- |
| Mental Health | Treatment of acute psychotic disorders | 58 | -130 | 57 | -128 | -2 | -- |
| NCDs | Diabetes Type II | 59 | -139 | 60 | -142 | 3 | -+ |
| Mental Health | Treatment of depression | 60 | -351 | 54 | -353 | 2 | -+ |
| Malaria | IPT (pregnant women) | 61 | -892 | 56 | -1,005 | 113 | -+ |
| RMNCH | Hypertensive disorder case management | 62 | -1,625 | 67 | -1,681 | 56 | -+ |
| RMNCH | Ectopic case management | 63 | -3,903 | 69 | -3,850 | -52 | -- |
| RMNCH | Post-abortion case management | 64 | -13,104 | 68 | -13,017 | -88 | -- |
| NCDs | COPD | 65 | -14,910 | 65 | -15,852 | 942 | -+ |
| RMNCH | Daily iron and folic acid supplementation (pregnant women) | 66 | -41,123 | 66 | -36,420 | -4,702 | -- |
| NCDs | Hypertension | 67 | -43,955 | 64 | -47,362 | 3,408 | -+ |
| IMCI | Zinc | 68 | -58,476 | 63 | -66,266 | 7,790 | -+ |
| IMCI | ORS | 69 | -61,064 | 62 | -65,956 | 4,892 | -+ |
| RMNCH | Antibiotics for treatment of dysentery | 70 | -179,032 | 71 | -181,618 | 2,586 | -+ |
| Vaccine Preventable Diseases | BCG vaccine | 71 | -218,883 | 70 | -215,464 | -3,419 | -- |
| Vaccine Preventable Diseases | Polio vaccine | 72 | -475,267 | 72 | -467,260 | -8,008 | -- |
| Vaccine Preventable Diseases | Pneumococcal vaccine | 73 | -620,201 | 73 | -629,787 | 9,586 | -+ |
| NPB: net population benefit  EDE: equally distributed equivalent  ∆EDE: Change in equally distributed equivalent  ++: positive net population benefit and positive equity impact  +-: positive net population benefit and negative equity impact  -+: negative net population benefit and positive equity impact  --: negative net population benefit and negative equity impact | | | | | | | |

# References

[MALAWI], N. S. O. N. 2018. 2018 Malawi Population & Housing Census. Zomba: National Statistical Office.

CROFT, T. N., MARSHALL, A. M. J. & ALLEN, C. K. A. 2018. Guide to DHS Statistics. Rockville, Maryland, USA: ICF.

DAWKINS, B. R., MIRELMAN, A. J., ASARIA, M., JOHANSSON, K. A. & COOKSON, R. A. 2018. Distributional cost-effectiveness analysis in low- and middle-income countries: illustrative example of rotavirus vaccination in Ethiopia. *Health Policy Plan,* 33**,** 456-463.

DE WALQUE, D. & FILMER, D. 2013. Trends and Socioeconomic Gradients in Adult Mortality around the Developing World. *Population and Development Review,* 39**,** 1-29.

JAGGER, C., COX, B. & LE ROY, S. 2006. Health expectancy calculation by the Sullivan Meothd. *Technical Report.* Montpellier: European Health Expectancy Monitoring Unit (EHEMU).

SULLIVAN, D. F. 1971. A single Index of Mortality and Morbidity. *HSMHA Health Reports,* 86**,** 347-354.

WHO 2014. WHO methods for life expectancy and healthy life expectancy. *Global Health Estimates Technical Paper WHO/HIS/HSI/GHE/2014.5.* Geneva: WHO.
